# The potential of platelet-rich plasma and PRP-derived biologics for neurological disorders: mechanisms and translational research

**DOI:** 10.3389/fimmu.2026.1858720

**Published:** 2026-09-08

**Authors:** Guina Tan, Xiaoxin Wang, Jing Chen, Ru Feng, Hongyu Chu, Degang Yang, Jun Li, Mingliang Yang, Ying Huang, Jianjun Li, Feng Gao

**Affiliations:** 1 School of Rehabilitation, Gannan Medical University, Ganzhou, Jiangxi, China; 2 Department of Spinal and Neural Functional Reconstruction, China Rehabilitation Research Center, Beijing, China; 3 Spinal Cord Injury Repair Laboratory, China Rehabilitation Science Institute, Beijing, China; 4 School of Rehabilitation, Capital Medical University, Beijing, China; 5 School of Rehabilitation, Wenzhou Medical University, Wenzhou, Zhejiang, China; 6 Department of Rehabilitation Medicine, Peking University Hospital, Beijing, China; 7 Cheeloo College of Medicine, Shandong University, Jinan, Shandong, China

**Keywords:** administration routes, axonal regeneration, neural repair, neuroinflammation, platelet-rich plasma (PRP)

## Abstract

Platelet-rich plasma (PRP), an autologous blood derivative rich in multiple growth factors and cytokines, has shown broad application prospects in the field of tissue repair and regeneration. In recent years, the therapeutic potential of PRP in neurological disorders has gained increasing attention. This article systematically reviews the classification systems, preparation methods, and the molecular mechanisms and application progress of PRP in neural repair. In preclinical studies, PRP has been shown to release insulin-like growth factor-1 (IGF-1), platelet-derived growth factor (PDGF), vascular endothelial growth factor (VEGF), and transforming growth factor-β (TGF-β), which may synergistically activate PI3K/Akt and MAPK/ERK signaling pathways, suggesting potential tissue repair, axonal regenerative, angiogenic, and immunomodulatory effects. The three-dimensional fibrin scaffold formed upon platelet activation not only provides physical support for cell migration and axonal growth but also enables the sustained local release of growth factors. In central nervous system disorders, intrathecal PRP has shown potential in animal models to alleviate neuroinflammation after spinal cord injury(SCI), and intranasal administration can improve cognitive function and neurogenesis in Alzheimer’s disease(AD) models. In peripheral nervous system disorders, local injection or PRP filling within nerve conduits significantly promotes regeneration and functional recovery after injuries to the sciatic nerve, facial nerve, and others. Furthermore, the therapeutic efficacy of PRP is influenced by factors such as preparation method, cellular composition (leukocyte content), administration route, and activation method. Although preclinical evidence is substantial, the clinical application of PRP in neurological disorders still faces challenges including a lack of standardization, unclear mechanisms, and the need to verify long-term safety. This review aims to provide a theoretical basis and practical reference for further research and clinical translation of PRP in the field of neural repair.

## 1 Introduction

PRP is an autologous platelet concentrate prepared primarily by centrifugation of whole blood, which increases the platelet concentration to 4 to 8 times the physiological baseline (1). Rich in growth factors, cytokines, and other bioactive mediators, it has expanded from orthopedics and dentistry into neural regeneration strategies. Through these components, PRP regulates key repair processes, including inflammation, angiogenesis, cell proliferation, and matrix remodeling (2, 3).

Experimental evidence suggests that PRP may improve the local reparative microenvironment, particularly during the early phase of tissue injury. By releasing bioactive mediators, PRP has been reported to attenuate inflammatory responses, reduce oxidative stress, inhibit apoptosis, support cell survival, and promote early regenerative events. For example, in a rabbit model of mechanically induced skeletal muscle ischemia, intramuscular PRP injection was associated with biochemical changes indicating reduced inflammatory activity and enhanced antioxidant defense. Histological analysis also showed less interstitial edema and reduced muscle fiber atrophy, suggesting a pro-reparative effect in the acute post-injury phase (4). However, the therapeutic effects of PRP are not uniform across different tissues or disease contexts. In a rabbit model of osteoarthritis, intra-articular PRP failed to prevent degenerative changes of the articular surface, whereas bone marrow aspirate concentrate showed better preservation of tibial hyaline cartilage thickness (5). These findings indicate that PRP may be more effective in certain acute or partially reversible injury settings, but may be insufficient to halt complex, chronic, or progressive degenerative processes. Similarly, clinical studies, especially in traumatology and orthopedics, have suggested potential benefits of PRP in pain relief, functional recovery, and tissue healing in selected indications, although outcomes remain heterogeneous because of differences in disease type, PRP formulation, activation method, and administration protocol (6–8).

Because these divergent therapeutic outcomes stem from the distinct biological activities of various PRP components, establishing a precise terminological framework is essential before evaluating disease-specific applications. PRP refers to the unmodified platelet-rich plasma obtained directly from autologous blood. By contrast, “PRP-derived products” refers to the bioactive components released from or isolated from PRP after platelet activation or further processing (9). These include the PRP secretome, which comprises the total pool of soluble mediators released by activated platelets, such as growth factors, cytokines, chemokines, and other signaling proteins (10). PRP-derived extracellular vesicles (EVs) are membrane-enclosed particles that carry proteins, lipids, and nucleic acids and mediate intercellular communication (11, 12). Within this broader EV category, exosomes (PRP-Exos) represent a specific small EV subtype generated through the endosomal pathway, they should therefore be understood as a subset of PRP-derived EVs rather than as an independent category (13). In addition, biomaterial-based formulations, such as hydrogels, fibrin matrices, or nerve guidance conduits loaded with PRP or its derivatives, are not PRP-derived products in a strict biological sense, but engineered delivery platforms designed to improve local retention, stability, and controlled release (14). Throughout this review, these terms are used consistently to distinguish the raw PRP product from its functional derivatives and delivery systems, thereby allowing the subsequent pathology-focused sections to concentrate on the relevant experimental and clinical evidence.

Applying this unified framework reveals that the therapeutic potential of PRP-based interventions diverges significantly across neurological conditions. Although the efficacy of PRP in repairing peripheral nerve injury (PNI) has been supported by a growing body of preclinical and clinical evidence (15–17), its application in more complex central nervous system (CNS) disorders—such as SCI and AD—remains at an earlier stage of investigation, with most studies confined to animal models (18, 19). CNS disorders are characterized by microenvironmental dysregulation, sustained inflammatory cascades, and irreversible neuronal loss (20). Moreover, the lack of standardized preparation protocols and a full understanding of the underlying molecular mechanisms continue to impede clinical translation. To overcome these constraints, combining PRP or its bioactive derivatives with advanced biomaterials has emerged as a promising approach to enhance local retention, ensure controlled release, and effectively modulate the hostile microenvironment of neural injuries.

Against this background, This review systematically evaluates the biological properties of PRP and its major derivatives, including the secretome and EVs, particularly PRP-Exos, and analyzes their multidimensional mechanisms of action in promoting neural repair through angiogenesis, immunomodulation, and anti-apoptotic pathways. Given the limitations of PRP as a stand-alone intervention, this review further focuses on the application of PRP in combination with biomaterials, such as three-dimensional hydrogel scaffolds, to improve the microenvironment of nerve injury. We also critically evaluate its translational potential and safety limitations across various neurological disorders, with the aim of providing a theoretical basis for the future development of precision PRP-based neurotherapies.

## 2 Fundamental principles and technical standardization of PRP

### 2.1 Classification systems for PRP

With the advancement of PRP research, its classification system has undergone an evolution from qualitative to quantitative, from single parameters to multidimensional parameters, and from fixed categories to modular nomenclature. In 2009, Dohan Ehrenfest et al. (21) first classified PRP into four categories based on the presence or absence of leukocytes and fibrin architecture (**Table 1**). However, this qualitative classification lacked quantitative measurements, making it difficult to account for the differences in tissue repair effects conferred by varying platelet concentrations, thereby limiting its guiding value in refined applications such as neural repair. In 2012, DeLong et al. (22) introduced the PAW system (**Table 1**), which incorporated three quantitative parameters—platelet absolute concentration, leukocyte content, and activation method—with exogenous activation denoted by “X” and endogenous activation left unmarked, thereby significantly enhancing the operability of the classification and comparability across studies. Similarly to DeLong et al, Mishra et al (23) also published a classification based in platelet concentration, the presence or absence of WBCs in the PRP, and the use of agonists to activate PRP (**Table 1**). However, the authors classified the variables in a different way to DeLong et al,9 identifying four types of PRP: 1) type 1 contains an increased concentration of platelets and WBCs over baseline, and is not activated by an exogenous activator such as thrombin or calcium; 2) type 2 contains both increased platelets and WBCs and is activated with thrombin and or calcium; 3) type 3 contains only an increased concentration of platelets without any WBCs and is not activated prior to application; and 4) type 4 contains only an increased platelet concentration and is activated with thrombin and/or calcium. Subtype A contains an increased platelet concentration at or above five times baseline. Subtype B contains an increased platelet concentration less than five times baseline. Nevertheless, the three preceding classifications do not take into account the final volume of the preparation delivered.

**Table 1:** Summary of published classifications for PRP.

Study	Platelet concentration	Leukocytes	Erythrocyte-s	Activation method	Centrifugat-ion steps	Preparation efficiency (platelet recovery)	Purity (platelet proportion)	Collection method	Image guidance	Light activation	Additional processing
Dohan Ehrenfest, D. M. et al. (2009) (21)	Not quantified	Present/absent	NR	Not specified	NR	NR	NR	NR	NR	NR	NR
DeLong, J. M. et al. (2012) (22)	P1–P4 categories P1 ≤ baseline P2 > baseline–750k/μL P3 >750k–1,250k/μL P4 >1,250k/μL	A above baseline B ≤ baseline α: neutrophi-l filtration	NR	X (exoge-nous activation)	NR	NR	NR	NR	NR	NR	NR
Mishra, A. et al. (2012) (23)	Fold increase A ≥5-fold baseline B <5-fold baseline	Present/absent (types 1/2 contain, 3/4 do not)	NR	Activated/not activated (types 2/4 activated)	NR	NR	NR	NR	NR	NR	NR
Mautner, K. et al. (2015) (24)	Absolute count (/μL)	+/- Neutrophil %	+/-	Yes/No	NR	NR	NR	NR	NR	NR	NR
Magalon, J. et al. (2016) (25)	Total dose categorie-s A >5×10^9^ B 3–5×10^9^ C 1–3×10^9^ D <1×10^9^	See “purity”	See “purity”	Yes/No	NR	A–D categorie-s A >90% B 70–90% C 30–70% D <30%	A–D categorie-s A >90% B 70–90% C 30–70% D <30%	NR	NR	NR	NR
Lana, J. F. S. D. et al. (2017) (26)	Fold increase 2–3/4–6/6–8/8–10 × baseline	R rich/P poor	R rich/P poor	A+ activated/A– not activated	SP1 (1 spin) SP2 (2 spins)	NR	NR	M automated/H manual	G+/G–	L+/L–	NR
Harrison, P. (2018) (27)	A/B categorie-s (high/low)	+/- (≥1% cutoff)	+/- (≥10% cutoff)	I not activated II activated	Implied in preparati-on type (1/2)	NR	NR	NR	NR	NR	NR
Acebes-Huerta, A. et al. (2020) (28)	Not directly included; reflected in naming; no unified threshold	Prefix L- (above PRP threshold)	Prefix Red-	Subscript A (thrombin/Ca^2+^) NT (induced release) Se (lysis)	Subscript can indicate (e.g., 1C, 2C)	Not directly included	Not directly included	Subscript Ap/BR (apheresis/whole blood)	Extensible subscript	Extensible subscript	Superscript (e.g., FT freeze-thaw, D debris removal)
Kon, E. et al. (2020) (29)	6-level fold increase (N1N2) 01: <2× 02: 2–3× 03: 3–4× 04: 4–5× 05: 5–6× 06: >6×	00: poor (≤ baseline) 01: rich (> baseline)	0: poor 1: rich	0: non-activated 1: exogenous activation	NR	NR	NR	NR	NR	NR	NR

Subsequently, the classification system further extended into the dimension of preparation processes. In 2015, Mautner et al. (24) established the PLRA system, incorporating erythrocyte content into the documentation requirements (**Table 1**). In 2016, Magalon et al. (25) proposed the DEPA system, which introduced two process parameters for the first time—”preparation efficiency” (platelet recovery rate) and “purity” (relative proportion of platelets in PRP) (**Table 1**). In 2017, the MARSPILL system incorporated key process variables such as automated versus manual preparation, centrifugation steps, image guidance, and photoactivation into the classification framework (26) (**Table 1**). In 2018, the International Society on Thrombosis and Haemostasis (ISTH) established a binary classification based on threshold values for leukocytes (≥1%) and erythrocytes (≥10%), providing a simplified approach for clinical quality control (27) (**Table 1**). However, this classification only dichotomizes platelet concentration as high or low, making it difficult to capture the refined dose-response relationships revealed by systems such as PAW or DEPA. In 2019, Gutiérrez et al. (28) proposed a modular nomenclature system (prefix–core–subscript–superscript) pursuing high extensibility, which in theory enables precise comparisons across different preparation protocols (**Table 1**). However, its complex naming rules have limited its adoption in routine clinical practice.

In 2020, Kon et al. (29) introduced a six-digit coding system (N1N2-N3N4-N5N6) that encodes platelet concentration (relative to baseline), leukocyte content, erythrocyte content, and activation status in a compact numerical format (**Table 1**). This system strikes a practical balance between the oversimplified ISTH binary classification and the overly complex Gutierrez modular nomenclature. By providing a standardized yet user-friendly coding scheme, it facilitates cross-study comparisons and has been increasingly adopted in clinical research on PRP for osteoarthritis and other musculoskeletal conditions.

In summary, the PRP classification system has evolved from early qualitative descriptions into a multidimensional parametric framework encompassing platelet dose, preparation efficiency, purity, leukocyte and erythrocyte content, activation method, and operational parameters. However, how to strike a balance between the precision of preparation protocols and the convenience of clinical application remains a central challenge yet to be resolved in the standardization process of PRP.

### 2.2 Preparation of PRP

PRP is not a single uniform product but a heterogeneous family of autologous platelet-derived preparations. From the perspective of preparation strategy and final composition, currently used approaches can be broadly classified into three major categories: (1) conventional centrifugation-based PRP, which usually results in relatively modest platelet enrichment; (2) platelet concentrates generated through serial concentration techniques, which achieve higher platelet concentrations; and (3) highly concentrated platelet products combined with fibrin, which may further modify structural properties and growth factor release kinetics. Recognizing these categories is important because differences in platelet concentration, leukocyte content, erythrocyte contamination, and fibrin architecture may substantially influence biological effects and limit comparability across studies.

Conventional centrifugation-based PRP remains the most traditional and widely used preparation strategy. Its basic principle involves the density-based separation of blood components by centrifugal force, with platelets being primarily enriched in the buffy coat. Depending on the number of centrifugation steps, it can be classified into single-spin and double-spin methods. The single-spin method typically employs centrifugation at 200-800g for 10-15 minutes. It is simple to operate and cost-effective, but achieves a platelet recovery rate of approximately 50%-65% and yields a relatively high leukocyte content (30, 31). By contrast, the double-spin approach uses an initial soft spin to isolate the platelet-containing plasma fraction, followed by a harder second spin to further concentrate platelets, thereby achieving higher recovery rates (31, 32). Commercial kits are essentially standardized variants of centrifugation-based preparation and offer operational simplicity and lower contamination risk through closed systems; however, their final platelet yield and cellular composition vary considerably among manufacturers, which complicates inter-study comparisons (33, 34).

A second major approach involves serial concentration techniques designed to generate platelet concentrates with higher platelet enrichment than conventional PRP. This category includes stepwise or repeated concentration protocols, as well as automated separation platforms such as standard cell separators and blood cell separators. Compared with conventional PRP, these methods generally provide more controlled and reproducible platelet collection, allow larger preparation volumes, and may reduce leukocyte contamination. Standard cell separators usually rely on continuous or intermittent centrifugation to isolate platelet-rich fractions in a relatively standardized fashion (35). Blood cell separators use software-controlled continuous blood flow to selectively collect platelets while returning the remaining blood components to the patient, making them suitable for the preparation of large-volume, leukocyte-poor PRP (36, 37). Platelet concentrations produced by blood cell separators have been reported to range from 1.4×10^6^/μL to 1.6×10^6^/μL, with leukocyte levels as low as 0.003×10³/μL (38). However, some devices may induce premature platelet activation due to shear stress during the separation process, thereby affecting product quality.

A third category consists of highly concentrated platelet products combined with fibrin. In these preparations, platelets are combined with a fibrin network or matrix, which may provide structural support, improve local retention, and prolong the release of platelet-derived bioactive factors (39). Compared with liquid PRP, fibrin-containing platelet products may therefore function not only as a source of growth factors but also as a provisional scaffold for tissue remodeling (40, 41). At the same time, their biological behavior, physical properties, and release kinetics differ from those of conventional PRP, meaning that results obtained with fibrin-enriched platelet products should not be directly equated with those of liquid PRP formulations (41).

In summary, the choice of PRP preparation method requires a trade-off between product quality and clinical operability. Centrifugation is cost-effective and operationally flexible, making it suitable for small-volume, individualized preparation. Commercial kits are convenient but exhibit high product heterogeneity. Automated separation equipment, although more costly, enables standardized production of large-volume, leukocyte-poor PRP. Future studies are needed to further clarify the impact of different preparation methods on the efficacy of neural repair, thereby guiding method selection in clinical practice.

### 2.3 Structural and regenerative biological effects of PRP

The biological rationale for PRP in nerve repair is grounded in its ability to provide a localized source of platelet-derived bioactive factors together with a provisional fibrin matrix at the injury site (16, 42). Because these mediators act predominantly through short-range interactions, PRP is most plausibly applied in anatomically accessible peripheral nerve and nerve root lesions, where the local microenvironment can be directly modified. Accordingly, this section focuses on the biological effects of PRP.

In nerve repair, PRP has been used in several established application formats, including direct placement at the coaptation or repair site, injection into the lumen of grafts or conduits, combination with nerve guidance tubes, and incorporation into fibrin-based matrices or scaffold systems (42–45). PRF represents a related formulation in which platelets are retained within a fibrin clot, providing a more stable local scaffold than liquid PRP (46). These formats are not biologically identical and may differ in retention, release kinetics, and mechanical support; however, they share the common objective of maintaining PRP-derived bioactive mediators at the repair interface.

During surgical nerve reconstruction, PRP may act as both a biological adjunct and a fibrin-based provisional matrix that supports local retention of regenerative mediators. By helping to preserve a pro-regenerative niche at the repair interface, PRP may promote Schwann cell activation and migration, facilitate Büngner band formation, and support axonal elongation across the lesion gap, with remyelination occurring as a later stage of successful nerve regeneration (47, 48). In addition, PRP may contribute to extracellular matrix organization and tissue integration at the repair site, thereby helping to stabilize structural continuity while limiting excessive fibrosis or scar formation that could otherwise impair axonal regrowth (49).

The biological rationale for PRP in peripheral nerve and nerve root disorders also extends beyond structural repair alone. These conditions are frequently associated with local inflammation, perineural adhesion, mechanical entrapment, and impaired microcirculation, all of which may compromise neural recovery. Experimental evidence suggests that PRP can modulate the inflammatory microenvironment by inhibiting NF-κB signaling, reducing the expression of pro-inflammatory mediators such as TNF-α and IL-1β, and promoting a shift in macrophage polarization toward a reparative M2 phenotype (50–52). In parallel, PRP-derived angiogenic factors, including VEGF and bFGF, may enhance endothelial activity and improve local microvascular support, thereby helping to alleviate the ischemic microenvironment surrounding the affected nerve (53). Through these combined actions, PRP may reduce inflammatory sensitization and neural irritability while creating conditions more favorable for structural preservation and functional recovery.

These considerations support a biologically plausible role for PRP in accessible peripheral nerve and nerve root lesions, where local application can directly modify the perilesional microenvironment. By contrast, extrapolation of this rationale to diffuse central neurodegenerative disorders remains much more uncertain (54). In such settings, the limitations are not only biological but also anatomical and translational, and should therefore be considered in relation to delivery issues discussed separately in Section 3.4. Overall, the currently available evidence supports a stronger biological rationale for PRP in localized peripheral nerve repair than in central nervous system disorders.

### 2.4 Immunological considerations for CNS administration: differences between L-PRP and P-PRP

After clarifying the various CNS administration routes of PRP, it is crucial to focus on the unique immunological risks associated with these routes, especially the key differences between L-PRP and P-PRP. PRP is not a single preparation, but a heterogeneous biological product significantly influenced by the preparation process. Its immunological effects are highly dependent on cellular composition, especially the content of white blood cells (55). Leukocyte-enriched PRP (L-PRP) typically contains a higher proportion of neutrophils and monocytes, which are important sources of various pro-inflammatory molecules, including interleukin-1β (IL-1β), tumor necrosis factor-α (TNF-α), interleukin-6 (IL-6), matrix metalloproteinase-9 (MMP-9), and reactive oxygen species (ROS) (56, 57). Conversely, pure PRP/leukocyte-poor PRP (P-PRP/LP-PRP) emphasizes the repair-promoting growth factors released by platelets, theoretically reducing additional inflammatory burden (56).

In the CNS microenvironment, these pro-inflammatory molecules can act on various target cells to trigger a cascade of inflammatory reactions. Microglia, as resident immune cells of the CNS, are highly sensitive to IL-1β and TNF-α, and can be activated to polarize toward the pro-inflammatory M1 phenotype, further releasing more inflammatory mediators to form a positive feedback loop (58, 59). Meningeal macrophages located in the meninges and choroid plexus, serving as the first line of defense for CNS immune surveillance (60), can also be directly activated by pro-inflammatory factors in L-PRP, disrupting the integrity of the blood-brain barrier and blood-cerebrospinal fluid barrier, and promoting the infiltration of peripheral immune cells (61).

Different CNS administration routes further determine the differences in the immune risk spectrum. Preclinical studies have explored intrathecal administration of PRP, in which the injectate is delivered into the cerebrospinal fluid and may distribute along the subarachnoid space and spinal cord surface (62). In this setting, leukocytes and pro-inflammatory mediators contained in leukocyte-rich PRP may come into direct contact with the spinal cord membranes, dorsal root ganglia, and spinal cord parenchyma, thereby posing a potential risk of neural irritation or tissue injury. By contrast, direct clinical evidence regarding the safety and consequences of intrathecal PRP exposure remains limited (63). Therefore, the leukocyte content should be regarded as a key safety variable rather than a secondary process parameter. For intranasal administration, the immune risks of L-PRP first appear at the nasal mucosa, an immunologically active interface. Pro-inflammatory factors can activate nasal mucosal epithelial cells and lamina propria immune cells, leading to mucosal edema and impaired barrier function (64). At present, however, direct evidence is lacking to support the notion that PRP or its derivatives can traverse the nasal mucosal barrier and enter the brain. Therefore, any potential nasal–brain effects should be regarded as hypothetical rather than established. More importantly, local inflammation in the nasal mucosa may transmit inflammatory signals through the olfactory pathway and associated immune responses, which could theoretically influence central nervous system-related processes; however, the extent and clinical relevance of such crosstalk remain to be verified (65). This nasal-brain immune crosstalk process introduces a new risk link of mucosal immune barrier for intranasal administration, despite avoiding the blood-brain barrier. Accordingly, the major concern for intranasal L-PRP administration is the local mucosal immune barrier rather than direct brain delivery. In contrast, PRP- or platelet-rich fibrin-based applications have been reported in clinically relevant local settings such as epidural patching, spinal epidural anti-fibrosis treatment, and cranial dural closure or skull base reconstruction, suggesting that their most realistic translational use lies in local tissue repair rather than transnasal brain delivery (66–68).

## 3 Molecular mechanisms underlying PRP-mediated nerve repair

The neural repair effects of PRP are attributed to its dual characteristics: functioning as a bioactive signaling reservoir rich in multiple growth factors, and forming a three-dimensional fibrin scaffold upon activation. These two properties act synergistically to construct a microenvironment conducive to neural repair by regulating multiple key biological processes, including tissue recovery, axonal regeneration, angiogenesis, and immunomodulation.

### 3.1 Repair-related effects and anti-apoptosis of PRP

The neuroprotective effects of PRP are primarily attributed to the synergistic activation of intracellular survival signaling pathways by its abundant growth factors. Among these, insulin-like growth factor-1 (IGF-1) and brain-derived neurotrophic factor (BDNF) are key effector molecules. Upon binding to IGFR-1/2 on the neuronal surface, IGF-1 activates the PI3K pathway, leading to Akt phosphorylation and its subsequent translocation to the nucleus, thereby inhibiting the expression of pro-apoptotic proteins such as Bax and caspase-3 and promoting neuronal survival (69, 70). In a mouse model of Alzheimer’s disease, intranasal administration of PRP significantly activated the PI3K/Akt signaling pathway, reduced Aβ-induced neuronal apoptosis, and upregulated the expression of heat shock protein HSP70, thereby exerting anti-apoptotic effects (19, 54). In addition, platelet-derived growth factor (PDGF) has also been shown to contribute to neuronal homeostasis and repair-related processes by modulating neuron-glial interactions (71, 72).

### 3.2 Promotion of axonal regeneration and Schwann cell activation

The core mechanism by which PRP promotes axonal regeneration lies in its direct regulation of Schwann cells. Schwann cells serve as the “engine” of peripheral nerve repair, and their capacity for proliferation, migration, and myelination directly determines the efficiency of axonal regeneration. Studies have shown that PDGF, fibroblast growth factor (FGF), and transforming growth factor-β (TGF-β) in PRP act synergistically to promote Schwann cell proliferation and migration (54). Zheng et al. further demonstrated that PRP induces Schwann cells to secrete neurotrophic factors (such as NGF and GDNF) in a dose-dependent manner, and upregulates the expression of genes associated with cell adhesion and axonal regeneration (73). In animal models, local application of PRP significantly increased the number of PCNA-positive Schwann cells, accompanied by improvements in regenerating axon diameter and myelin sheath thickness (74). In addition, IGF-1 and VEGF exhibited synergistic effects on axonal growth in a brain-spinal cord coculture system, and neutralizing these two factors significantly attenuated the pro-regenerative effect of PRP, suggesting that they are key molecules mediating PRP-induced axonal regeneration (75).

### 3.3 Induction of angiogenesis and microenvironment remodeling

Axonal regeneration is highly dependent on the oxygen and nutrients supplied by newly formed blood vessels. The high concentration of vascular endothelial growth factor (VEGF) in PRP serves as the core driver of angiogenesis. Upon binding to VEGFR2 on the surface of vascular endothelial cells, VEGF activates the PLCβ-IP3/DAG-PKC cascade, promoting endothelial cell migration and lumen formation (76). In models of nerve injury, PRP not only directly induces angiogenesis through VEGF but also provides physical support for new vessels via the fibrin scaffold, forming “vascular tracks” conducive to axonal extension (77). Kim et al. demonstrated in a rat sciatic nerve defect model that early angiogenesis was significantly enhanced in vein grafts filled with PRP, and this enhancement showed a spatial correlation with axonal regeneration (43). In addition, VEGF indirectly supports neuronal survival and regeneration by inhibiting microglial proliferation and alleviating neuroinflammation (78).

### 3.4 Regulation of neuroinflammation and the immune microenvironment

Excessive or persistent inflammatory responses following nerve injury represent a critical barrier to regeneration. The anti-inflammatory effects of PRP are primarily mediated through two pathways: first, transforming growth factor-β (TGF-β) suppresses the expression of pro-inflammatory cytokines (such as TNF-α and IL-1β) by inhibiting the NF-κB signaling pathway; second, hepatocyte growth factor (HGF) synergizes with TGF-β to induce macrophage polarization from the pro-inflammatory M1 phenotype toward the anti-inflammatory M2 phenotype (52, 79). In a spinal cord injury model, intrathecal administration of PRP significantly reduced the expression of GFAP, HMGB1, and PKM2 in the spinal dorsal horn—markers reflecting astrocyte activation, inflammatory mediator release, and metabolic reprogramming, respectively—suggesting that PRP effectively suppresses neuroinflammation in the central nervous system (62). *In vitro* studies have shown that PRP treatment completely blocks Aβ-induced expression of inflammatory factors in astrocytes, a mechanism also involving the inhibition of the NF-κB pathway (19).

Collectively, these findings indicate that PRP-mediated neural repair is achieved through multiple interconnected mechanisms rather than a single dominant pathway. Neuroprotective and anti-apoptotic signaling, Schwann cell activation, angiogenesis, and immune modulation act in parallel and reinforce one another within the injured tissue. In this context, the therapeutic activity of PRP depends not only on the identity of individual growth factors, but also on the coordinated local presentation of these signals over time. This consideration is particularly important when moving from whole PRP toward more defined cell-free fractions and delivery-assisted formulations.

### 3.5 PRP-derived secretome and bio-scaffolds

From a mechanistic perspective, the biological activity of PRP is not solely determined by the presence of intact platelets, but also by the spectrum of soluble mediators and extracellular vesicles released upon platelet activation. In this review, PRP-derived secretome refers broadly to these cell-free bioactive components, including soluble growth factors, cytokines, chemokines, and platelet-derived extracellular vesicles, particularly exosomes. These secreted factors constitute an important paracrine basis for the effects described in the preceding sections, as they collectively participate in the regulation of neuronal survival, angiogenesis, inflammatory modulation, and tissue remodeling (48, 80, 81).

Within this secretome, PRP-derived exosomes may represent a functionally concentrated fraction for intercellular signaling. By carrying proteins, lipids, mRNAs, and microRNAs, these nanoscale vesicles can protect bioactive cargo and facilitate its transfer to recipient cells (82). Accordingly, PRP-Exos may serve as a cell-free mediator capable of reproducing, at least in part, the neuroprotective, anti-apoptotic, pro-angiogenic, and immunomodulatory properties traditionally associated with PRP (83, 84). This feature is particularly relevant when considering the mechanistic continuity between whole PRP and more defined PRP-based derivatives.

However, the therapeutic efficiency of secretome-derived signals depends not only on their biological potency but also on their spatiotemporal presentation at the injury site. Rapid diffusion, limited local retention, and post-administration instability may all restrict the persistence of these effects. In this context, bio-scaffolds function as a complementary platform for improving the delivery of PRP-derived factors and vesicles (44, 85). By providing a supportive matrix for local administration, hydrogel- and scaffold-based systems can enhance lesion-site retention, protect labile cargos, and enable sustained release, thereby prolonging exposure to neurotrophic and angiogenic cues (42, 86). This delivery function is mechanistically relevant to neural repair. A suitable scaffold may reinforce the regenerative actions of PRP-derived secretome not only by increasing local bioavailability, but also by providing structural support for cell infiltration, matrix remodeling, vascularization, and guided tissue integration (87). In this sense, scaffold-assisted delivery helps translate short-lived paracrine signaling into a more stable regenerative microenvironment. Materials such as alginate, GelMA, chitosan-based systems, and PEG-based hydrogels have therefore been explored as candidate platforms because they combine biocompatibility with injectability, lesion conformity, and controllable degradation (88).

Taken together, PRP-derived secretome and bio-scaffolds should be viewed as two functionally integrated dimensions of PRP-based regenerative engineering: the former supplies the principal bioactive signals, whereas the latter improves their localization, persistence, and therapeutic presentation. Rather than representing concepts separate from the molecular mechanisms outlined above, they extend those mechanisms toward more controllable and translationally relevant intervention formats, thereby providing a bridge to the pathology-specific applications discussed in the following sections.

## 4 Treating neurological disorders with PRP therapy

Currently, extensive preclinical research and clinical trials have explored the application of PRP therapy in neurological disorders. By leveraging the functional or physiological properties of PRP, it holds promise for reducing neuroinflammation, promoting neuronal survival and functional recovery, thereby improving patients’ clinical symptoms and quality of life. The following sections will elaborate on therapeutic strategies for PRP in neurological diseases.

### 4.1 Central nervous system disorders

The multifaceted regenerative properties of PRP—including tissue repair, axonal regeneration, angiogenesis, and immunomodulation—provide a rationale for its application in CNS disorders. Building on the mechanistic insights discussed in the previous section, this subsection focuses on three CNS conditions for which preclinical evidence has demonstrated therapeutic potential: SCI, AD, and Ischemic stroke (IS).

#### 4.1.1 Therapeutic potential of PRP in SCI

SCI, as a catastrophic neurological condition, often results in permanent sensory, motor, and autonomic dysfunction, imposing a significant personal and socioeconomic burden (89). The pathophysiological mechanisms of SCI are highly complex, involving not only primary mechanical trauma but also triggering a cascade of secondary injuries. These include ischemia, neuroinflammation, disruption of the blood-spinal cord barrier (BSCB), and the formation of a microenvironment that severely inhibits endogenous repair (90). Given its rich content of growth factors, cytokines, and other bioactive molecules, PRP has emerged as a potential regenerative strategy for SCI in preclinical research. However, the available evidence remains heterogeneous in terms of model system, PRP formulation, route of delivery, treatment regimen, and study endpoint. Therefore, the current literature requires critical interpretation rather than simple aggregation of positive findings. Importantly, direct clinical evidence supporting PRP-mediated neural repair in human SCI remains absent, and the existing supportive data are currently derived mainly from *in vitro* and animal studies.

**Figure 1:**
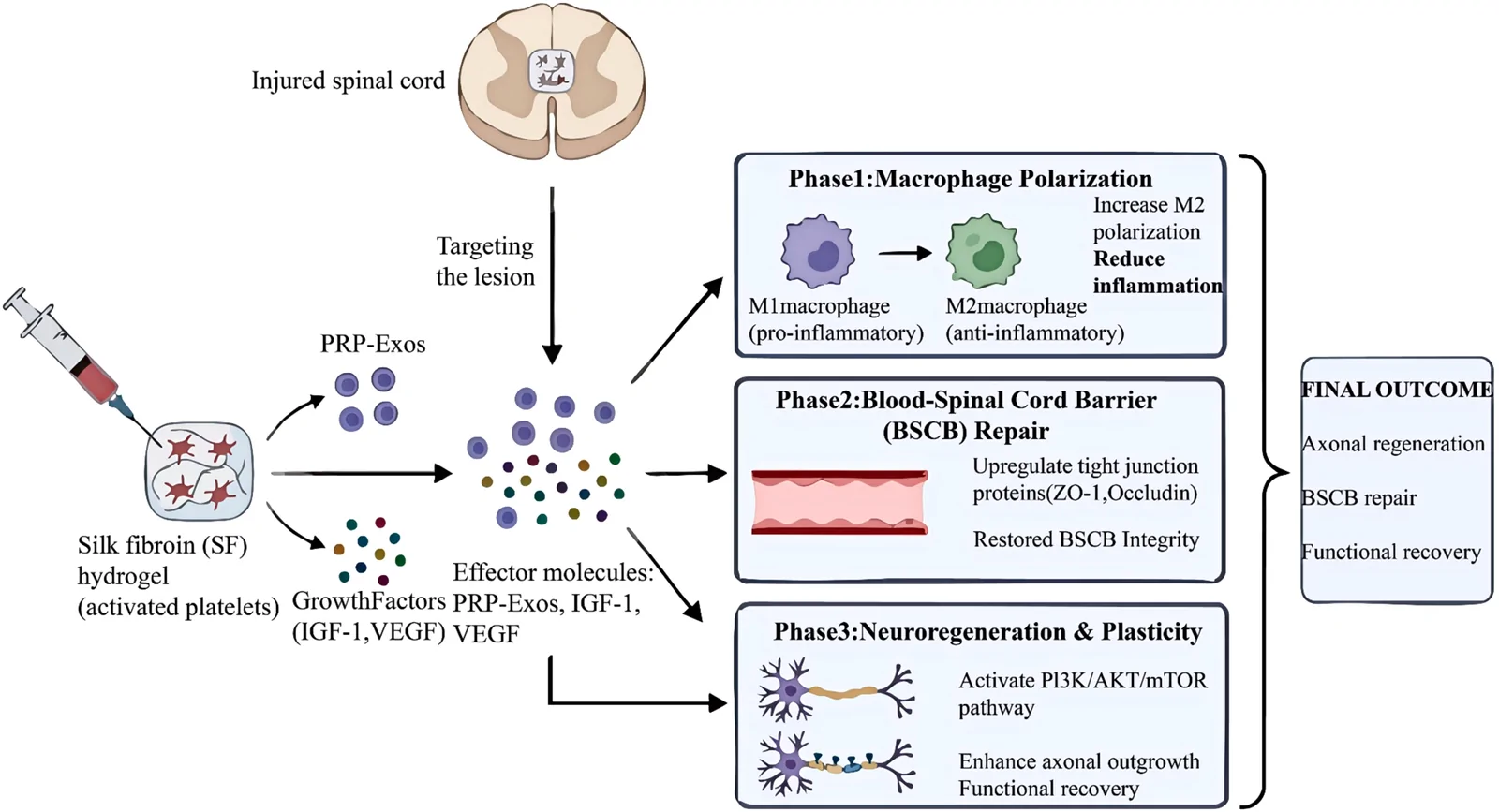
Schematic diagram illustrating the mechanisms of PRP and PRP-Exos in the treatment of spinal cord injury. Following spinal cord injury, fibrin hydrogels loaded with activated platelets and PRP-derived exosomes (PRP-Exos) are applied to the injured site, releasing bioactive molecules (IGF-1, VEGF). These bioactive molecules exert their reparative effects through three phases: Phase 1: Macrophage polarization (inhibiting M1-type and promoting M2-type polarization to reduce inflammation); Phase 2: Blood-spinal cord barrier repair (upregulation of tight junction proteins ZO-1 and Occludin, restoration of barrier integrity); Phase 3: Neural regeneration and plasticity (activation of the PI3K/AKT/mTOR pathway, enhancement of axonal growth). The ultimate outcomes are axonal regeneration, repair of the blood-spinal cord barrier, and functional recovery.

At the preclinical *in vitro* level, researchers have used brain–spinal cord co-culture models to investigate the potential molecular mechanisms underlying the effects of PRP (75). The study suggested that the pro-axonal effects of PRP were associated, at least in part, with IGF-1- and VEGF-related signaling (75). However, a critical evaluation of these findings highlights a notable molecular discrepancy: while regenerative factors promote growth, other co-existing components like TGF-β1 appeared to exert an inhibitory effect on axonal growth under the conditions tested and may also contribute to a less permissive reparative environment (75). Furthermore, although this simplified *in vitro* model is useful for exploring specific signaling pathways, it cannot reproduce the physical barriers, glial-scar dynamics, vascular changes, and systemic inflammatory responses present in the injured spinal cord *in vivo* (**Figure 1**).

Moving from cell cultures to *in vivo* animal models, preclinical research has primarily relied on rodent models of spinal injury to evaluate functional recovery (18). Study show that PRP treatment was associated with improved functional outcomes, increased neovascularization, and better preservation of white matter integrity (18). Methodologically, contrasting delivery kinetics reveals that sustained, low-dose administration regimens yield superior outcomes compared to single bolus injections, suggesting that sustained exposure and prolonged modulation of the local microenvironment may be more beneficial than a single bolus administration (18). However, these preclinical conclusions must be tempered with caution. Rodent models differ from human SCI in injury scale, spinal cord anatomy, lesion heterogeneity, chronicity, and the extent of spontaneous functional compensation. Consequently, the anatomical and locomotor recovery observed in these highly controlled animal models cannot be assumed to guarantee equivalent clinical efficacy in human patients (**Figure 1**).

Because the effects of soluble PRP factors may be limited by their short residence time and rapid dispersal in the injured spinal cord, preclinical research has shifted toward multi-component engineering platforms that pair PRP with stem cells and biomaterials. When combined with adipose-derived mesenchymal stem cells (AD-MSCs), PRP may provide a trophic and supportive microenvironment for AD-MSCs, suppressing cell apoptosis and accelerating axonal regeneration more effectively than either treatment alone (91). To address the physical challenge of cell retention at the injury site while potentially supporting the survival, integration, and neural differentiation of transplanted cells, subsequent researchers transitioned to structured biomaterials and neurogenic stem cell lines. By incorporating human dental pulp stem cells (hDPSCs)—which possess high intrinsic neurogenic potential—into a solidifying PRP hydrogel carrier, investigators improved stem cell survival and integration, leading to reduced cystic cavity formation and marked motor recovery in rat models (92). Furthermore, combining PRP with BDNF-overexpressing BMSCs has been shown to drive astrocyte migration and axonal remyelination (93) (**Figure 1**). While these biomaterial-based systems may partially address the limited retention and mechanical support of soluble PRP, they also introduce significant methodological complexity, making it difficult to isolate the precise therapeutic contribution of each component or standardize manufacturing protocols.

Taken together, current preclinical evidence suggests that the most plausible contribution of PRP in SCI is microenvironmental modulation rather than a definitively established direct regenerative effect. Across studies, its proposed functions include supporting angiogenesis, limiting secondary damage, and improving the local conditions for transplanted cells or biomaterial systems. At the same time, the evidence base remains limited and heterogeneous, with major variation in PRP formulation, dosing regimen, route of administration, injury model, and outcome assessment. These are not merely technical differences; they are likely determinants of biological activity and may account for much of the variability in reported benefit. Accordingly, PRP should currently be regarded as a promising but investigational strategy in SCI. Preclinical data support further study, particularly in well-controlled combinational settings, but they do not yet justify strong conclusions regarding clinical efficacy or optimal therapeutic use in human patients.

#### 4.1.2 Therapeutic potential of PRP in AD

AD is characterized by progressive neurodegeneration, chronic neuroinflammation, amyloid-β accumulation, tau pathology, and progressive cognitive decline (94). Platelets have increasingly been implicated in AD-associated inflammatory processes through their interactions with immune cells and the release of bioactive mediators (95–97). These observations have provided an indirect and exploratory rationale for investigating PRP as a potential therapeutic approach. However, the rationale for using PRP in AD remains exploratory, and the available evidence is currently limited to *in vitro* and animal studies, with no established clinical evidence supporting its efficacy or safety in patients with AD.

**Figure 2:**
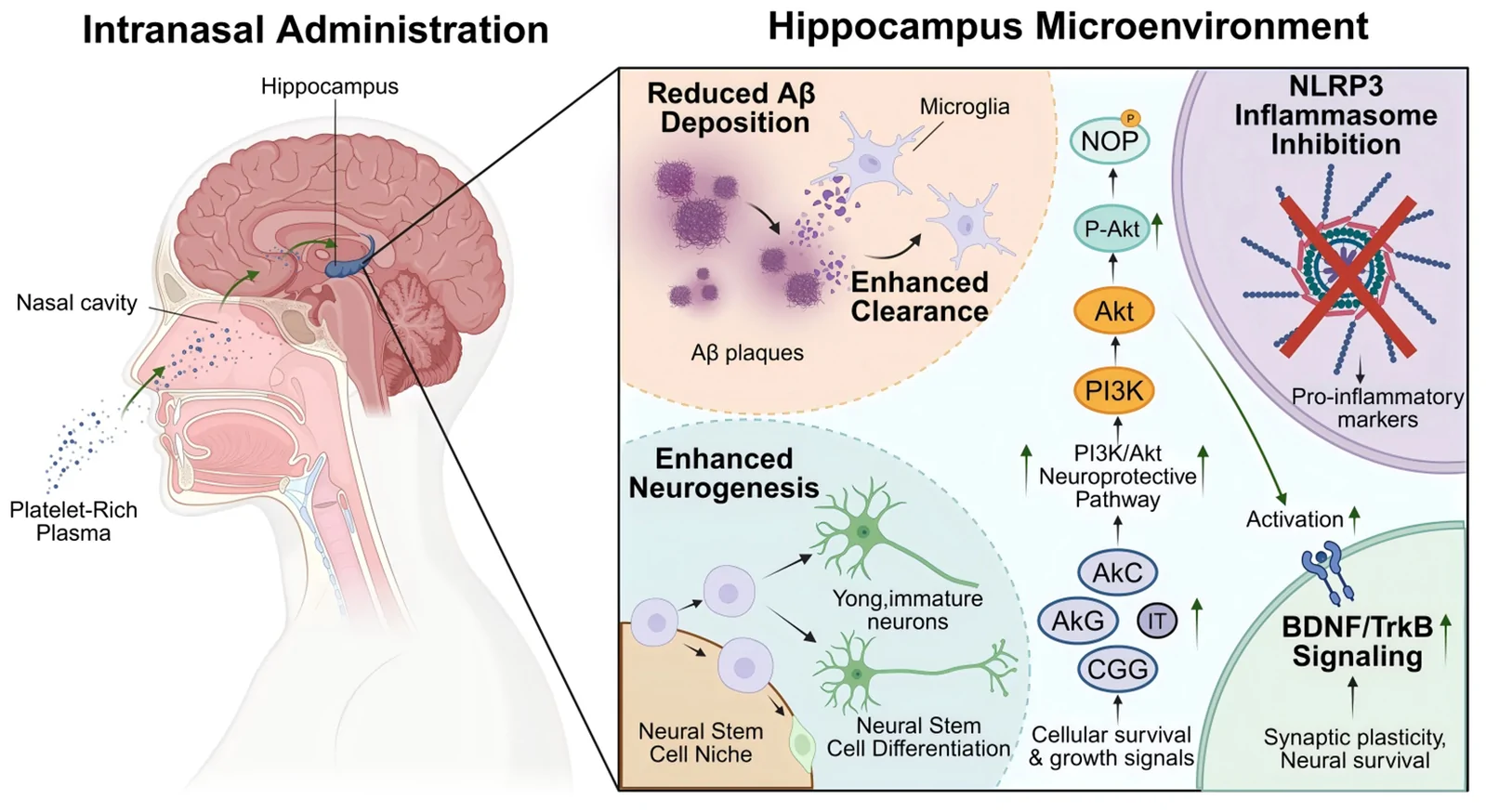
Schematic illustration of the neuroprotective mechanisms of intranasally administered Platelet-Rich Plasma (PRP) targeting the hippocampus microenvironment. (Left) Intranasal administration provides a direct, non-invasive nose-to-brain delivery route, allowing PRP to bypass the blood-brain barrier and specifically target the hippocampus. (Right) Within the hippocampal microenvironment, PRP-derived cellular survival and growth signals activate the central PI3K/Akt neuroprotective pathway. This master regulatory signaling cascade subsequently exerts multidimensional therapeutic effects through four key mechanisms: 1) Reduced Aβ Deposition: Enhancing microglial phagocytosis and clearance of Amyloid-beta (Aβ) plaques; 2) NLRP3 Inflammasome Inhibition: Suppressing NLRP3 inflammasome assembly to downregulate pro-inflammatory markers and mitigate neuroinflammation; 3) Enhanced Neurogenesis: Promoting the differentiation of neural stem cells from their niche into young, immature neurons to facilitate tissue repair; 4) BDNF/TrkB Signaling Activation: Upregulating the BDNF/TrkB pathway to enhance synaptic plasticity and promote overall neural survival.

A review of the limited preclinical literature reveals substantial methodological heterogeneity, which complicates cross-study comparison and limits assessment of the reproducibility of the reported effects of PRP. In APP/PS1 transgenic mice, which model selected aspects of amyloid-β pathology through the overexpression of AD-related transgenes, intranasal PRP administration was associated with increased hippocampal neurogenesis, reduced amyloid deposition, and modulation of PI3K/Akt signaling (98, 99). In contrast, in an STZ-induced model intended to reproduce selected features of sporadic AD, intravenous PRP administration did not produce the beneficial effects reported in the APP/PS1 studies and was associated with adverse pathological changes under the conditions tested (100). These discrepant findings highlight the context dependence of PRP responses and suggest that the disease model, delivery route, PRP formulation, dosing regimen, and outcome measures may all influence the observed effects. Intranasal administration may partially reduce BBB-related limitations through nose-to-brain transport (101, 102), whereas intravenous administration is influenced by systemic distribution, platelet clearance, and the extent to which PRP-derived factors can reach the central nervous system across the BBB (103). Moreover, APP/PS1 mice and STZ-induced models capture different aspects of AD-related pathology. APP/PS1 mice primarily model amyloid-β accumulation associated with transgene expression, whereas STZ treatment is used to induce selected metabolic, inflammatory, and cognitive abnormalities (100). Therefore, the beneficial effects observed in APP/PS1 mice may be model-dependent and cannot be assumed to represent a disease-modifying effect across the broader spectrum of human AD (**Figure 2**).

Mechanistic support for PRP has also been inferred from studies in other neurological models, in which PRP administration was associated with improved learning and memory and with modulation of the NLRP3 inflammasome or the BDNF/TrkB pathway (98, 99, 104) (**Figure 2**). Although these studies provide useful insights into potential molecular pathways, they should be regarded as indirect mechanistic support rather than direct evidence of efficacy in AD. Models of traumatic brain injury or chemical neurotoxicity generally do not reproduce the chronic and interacting amyloid-β and tau pathologies that characterize human AD (105, 106). Consequently, extrapolating broad neuroprotective mechanisms from these models to AD may oversimplify the therapeutic challenge and may not adequately account for the chronic, multifactorial, and heterogeneous pathological microenvironment of the AD brain.

Overall, the current evidence is insufficient to establish the efficacy or safety of PRP as a treatment for AD. The limited translation of many AD interventions from rodent models to human trials reflects multiple factors, including differences in disease duration, pathological heterogeneity, cognitive complexity, pharmacokinetics, treatment timing, and outcome assessment between experimental models and patients. Before meaningful clinical evaluation can be pursued, several important translational barriers must be addressed. These include characterizing the bioactive components of PRP and determining how their potentially divergent effects influence AD-related pathology, standardizing PRP preparation to improve reproducibility, and evaluating dose-, route-, and formulation-specific safety profiles in relevant preclinical models. Until these variables are better characterized and standardized, PRP should be regarded as an investigational approach in AD rather than an established therapeutic option.

#### 4.1.3 Therapeutic potential of PRP in IS

Neural repair after ischemic stroke remains challenging because the eligibility window for reperfusion therapies is limited, while excitotoxicity, oxidative stress, and neuroinflammation initiated during ischemia may persist or intensify during reperfusion, contributing to ongoing neuronal injury (107, 108). These limitations underscore the need for adjunctive strategies that could support tissue preservation, repair, and remodeling beyond the narrow window available for reperfusion therapy. In preclinical studies, PRP and PRP-EVs have shown preliminary potential to improve functional outcomes after ischemic stroke, possibly through modulation of inflammatory and oxidative-stress responses, neurogenic signaling, and vascular remodeling (109, 110). However, the relative contributions of whole PRP and its EV component remain incompletely defined.

**Figure 3:**
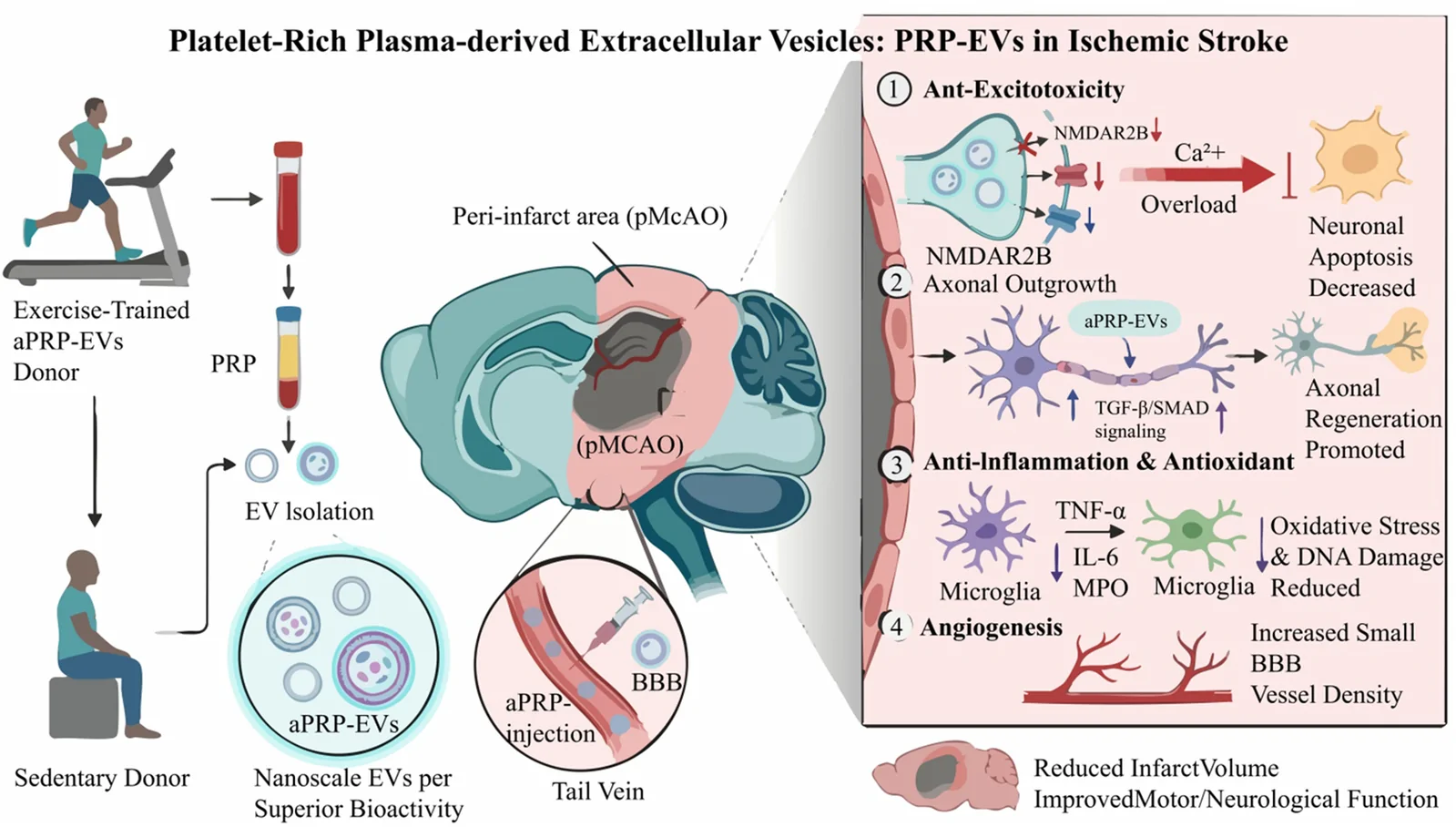
Schematic illustration of the therapeutic mechanisms of exercise-conditioned aPRP-EVs in ischemic stroke. Activated platelet-rich plasma-derived extracellular vesicles (aPRP-EVs) isolated from exercise-trained donors exhibit superior bioactivity compared to those from sedentary donors. Following tail vein injection, these nanoscale EVs successfully cross the blood-brain barrier (BBB) and target the peri-infarct area in a permanent middle cerebral artery occlusion (pMCAO) model. The aPRP-EVs exert pleiotropic neuroprotective effects through four distinct pathways: (1) Anti-excitotoxicity: Attenuating NMDAR2B overactivation and subsequent intracellular Ca2+Ca2+ overload, thereby decreasing neuronal apoptosis; (2) Axonal Outgrowth: Promoting axonal regeneration via the activation of TGF-ββ/SMAD signaling; (3) Anti-inflammation & Antioxidant: Modulating microglia to downregulate pro-inflammatory factors (TNF-αα, IL-6, MPO) and reduce oxidative stress and DNA damage; (4) Angiogenesis: Increasing small vessel density to support tissue repair. Ultimately, these multidimensional mechanisms synergistically lead to a reduced infarct volume and significantly improved motor and neurological function.

Evidence from animal models suggests that the effects of whole PRP may depend partly on the route of administration and may involve both central and peripheral responses. In a permanent ischemia model without reperfusion, intraventricular administration of PRP preparation was associated with reduced infarct volume and improved motor performance in rats (111). Because reperfusion was not performed, these findings suggest that PRP-derived factors may exert protective effects even in the absence of restored blood flow. However, the invasiveness of intraventricular administration, together with the associated risks of hemorrhage, infection, and procedural variability, may substantially limit its feasibility for clinical translation. In other preclinical studies, intra-arterial administration of PRP through vessels such as the carotid artery or abdominal aorta was associated with smaller infarct volumes and better neurological outcomes, including in models complicated by systemic organ injury (111, 112). These effects were accompanied by lower levels of inflammatory mediators, including TNF-α, IL-6, and MPO, in peripheral tissues and/or the brain, as well as by evidence of microvascular remodeling near the infarct. However, because these studies used specialized injury models and different intra-arterial delivery routes, the extent to which the findings apply to uncomplicated human ischemic stroke remains uncertain (112) (**Figure 3**). These findings raise the possibility that PRP acts through coordinated effects on inflammatory, vascular, and tissue-repair processes in both the brain and peripheral organs.

Although the aforementioned molecular and cellular mechanisms reveal the regenerative potential of PRP and its EVs, the gap between animal models and clinical translation remains vast. First, regarding “compositional equivalence and standardization of preparation,” it remains unclear whether purified EVs can fully substitute for whole PRP therapy, which is rich in complex matrices and factors; Second, current evidence of therapeutic efficacy relies heavily on rodent models that are young, healthy, and free of comorbidities. This represents a significant disconnect from the complex pathophysiological backgrounds—such as advanced age, hypertension, and diabetes—that often accompany clinical stroke patients (i.e., the presence of persistent microenvironmental imbalances and vascular aging).

### 4.2 Peripheral nervous system disorders

PNI is a common traumatic or iatrogenic neurological disorder (113). Its core pathophysiology involves axonal rupture and Wallerian degeneration, Schwann cell dedifferentiation and dysfunction, intra-neural inflammation and macrophage infiltration, as well as secondary fibrosis, scar formation, and atrophy of the target muscles (114, 115). These pathological changes collectively create a microenvironment that impedes effective neural regeneration. This section focuses on three categories of peripheral nerve disorders where PRP has demonstrated therapeutic potential: sciatic nerve injury, facial nerve injury, and other peripheral nerve injuries.

#### 4.2.1 The role of PRP in the repair of sciatic nerve injuries

Sciatic nerve injury is a form of PNI that may result from trauma, compression, traction, ischemia, or iatrogenic damage and is commonly associated with motor and sensory deficits (116). Its typical pathological processes include Wallerian degeneration following axonal transection, the formation of Büngner bands through Schwann cell dedifferentiation and proliferation, and the subsequent extension of regenerating axons along with myelin remodeling (117). Axonal regeneration may be limited when persistent inflammation, insufficient neurotrophic support, fibrotic scar formation, or other physical barriers create a nonpermissive regenerative microenvironment. This limitation may contribute to muscle denervation atrophy, neuropathic pain, and incomplete functional recovery (118). Therefore, restoring a microenvironment that supports axonal growth and Schwann-cell function may be an important, but not the sole, determinant of functional recovery after sciatic nerve injury.

**Figure 4:**
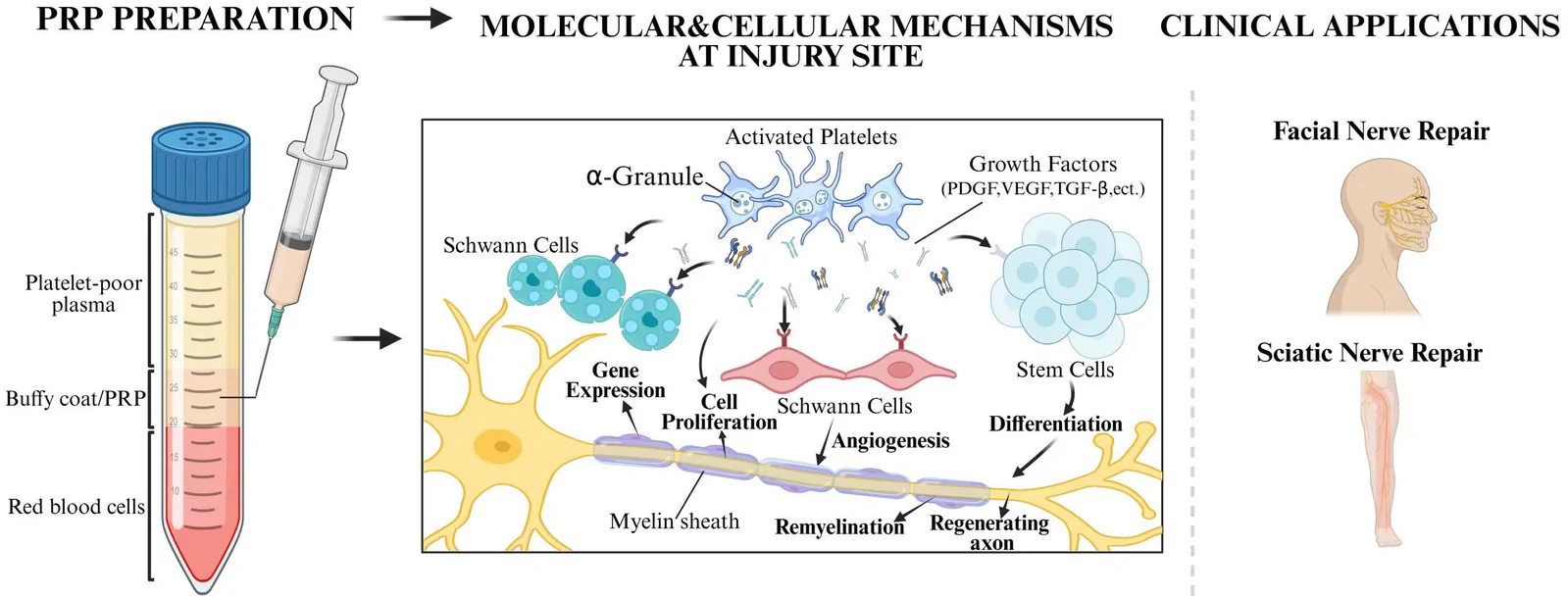
Schematic illustration of the translational workflow of Platelet-Rich Plasma (PRP) for peripheral nerve repair: from preparation to clinical application. (Left) PRP Preparation: Whole blood is processed via centrifugation, separating into three distinct layers: platelet-poor plasma at the top, red blood cells at the bottom, and the critical intermediate “buffy coat” containing the PRP, which is subsequently extracted via a syringe. (Middle) Molecular & Cellular Mechanisms: At the site of peripheral nerve injury, activated platelets undergo degranulation, releasing αα-granules packed with essential growth factors (e.g., PDGF, VEGF, TGF-ββ). These paracrine factors orchestrate a multidimensional regenerative microenvironment by acting on target cells: (1) stimulating Schwann cell proliferation and upregulating gene expression to facilitate remyelination; (2) inducing local angiogenesis to restore blood supply; and (3) promoting stem cell differentiation. Together, these synergistic cellular responses robustly support the outgrowth of the regenerating axon. (Right) Clinical Applications: Based on its potent regenerative mechanisms, PRP therapy is highly applicable in clinical settings for the treatment of peripheral nerve injuries, notably including facial nerve repair and sciatic nerve repair.

In preclinical studies exploring the therapeutic mechanisms of PRP, this therapy has demonstrated the ability to modulate the microenvironment through multiple targets and pathways. High-throughput proteomics analysis indicates that PRP may regulate angiogenesis and proteome homeostasis through multiple targets and pathways. Specifically, PRP intervention significantly upregulates the expression of integrin β-8 (ITGB8), thereby promoting local neovascularization via the focal adhesion signaling pathway. In addition, PRP regulates key targets such as ribosomal protein S27a (RPS27A) and ubiquilin-like protein 1 (UBQLN1), helping maintain intracellular proteome homeostasis through the ubiquitin system and laying the metabolic foundation for the survival of damaged neurons (15). In addition to regulating intracellular homeostasis, PRP can directly activate Schwann cells, the core supporting cells of the peripheral nervous system. This activation stimulates Schwann-cell proliferation, induces phenotypic conversion, and promotes the release of key neurotrophic factors (119). PRP gel has also been shown to relieve neuropathic pain caused by peripheral nerve injury by suppressing the expression of pro-inflammatory cytokines TNF-α and IL-6 at the injury site (118) (**Figure 4**). However, existing studies exhibit significant methodological heterogeneity, employing diverse experimental systems such as *in vivo* proteomics, *in vitro* cell culture, and *in vivo* pain models, which makes it challenging to quantitatively compare the actual relative contributions of each pathway to nerve regeneration (**Figure 4**).

With the gradual elucidation of preclinical mechanisms, the clinical translational value of PRP in treating sciatic nerve injury has also been preliminarily validated. Randomized controlled clinical trials have shown that combining ultrasound-guided multipoint PRP injections with standardized rehabilitation training can lead to significant and sustained improvements in nerve conduction and related functional outcomes in patients with incomplete sciatic nerve injury. These findings support the practical value of PRP delivery via minimally invasive intervention (116). To ensure precise measurement of this clinical efficacy and to guide subsequent protocol optimization, clinical evaluation systems are evolving from single-parameter assessments to multidimensional evaluations. Traditional measurements of single electrophysiological parameters may no longer comprehensively reflect the actual functional quality of nerve regeneration. New evaluation paradigms are therefore moving toward a multimodal integrated assessment system that combines gait trajectory analysis, video-based dynamic kinematic evaluation, static postural assessment, and histological verification (120). This composite endpoint evaluation paradigm, which combines patients’ macroscopic motor performance with microscopic biological indicators, not only enhances the scientific rigor of efficacy assessment but also provides more rigorous standards for protocol design in clinical research on peripheral nerve repair.

#### 4.2.2 The role of PRP in the repair of facial nerve injuries

Peripheral facial nerve palsy is a severe debilitating disorder that substantially impairs patients’ quality of life. Due to limited functional recovery during the chronic stage, prolonged denervation and irreversible target muscle atrophy may occur, resulting in permanent sequelae (121). Although microsurgical techniques, including epineurial neurorrhaphy and fascicular repair, remain the primary approaches for facial nerve reconstruction in clinical practice, their ability to fully restore axonal continuity and prevent long-term target muscle atrophy remains limited (122). Combining PRP therapy with facial nerve anastomosis has been proposed as a potential adjunctive strategy to enhance functional recovery; however, its clinical efficacy remains to be validated in well-designed human studies (123).

Preclinical studies suggest that this combined approach may facilitate structural and functional recovery of the facial nerve through multiple mechanisms, including neurotrophic support, modulation of inflammatory responses, and promotion of supporting cell proliferation. First, in the microenvironmental remodeling achieved by “PRP combined with stem cells,” PRP may function as both a biological stimulant and a delivery matrix by supporting the survival and paracrine activity of adipose-derived stem cells or neuroinduced MSCs, while promoting their partial acquisition of Schwann cell-like characteristics. When used in combination with decellularized allogeneic nerve grafts, this strategy has shown promising regenerative effects in experimental models of long-segment facial nerve defects (123, 124). Second, PRP-loaded chitosan hydrogels may prolong the local release of growth factors and provide structural support for axonal extension. Experimental studies have reported improvements in electrophysiological recovery and histological regeneration after facial nerve injury (125). Mechanistic studies suggest that PRP-derived growth factors may contribute to tissue repair and axonal regeneration by enhancing Schwann cell activity, supporting neuronal survival, and promoting distal axonal growth after nerve injury (126). These neuroprotective effects have been reported in several models of traumatic or compressive facial nerve injury; however, the extent to which these findings can be translated into clinically meaningful functional recovery remains uncertain (**Figure 4**).

Although *in vitro* and animal studies have provided accumulating evidence supporting the neurotrophic potential of PRP in facial nerve regeneration, these findings should be interpreted cautiously because experimental conditions differ substantially from clinical scenarios. On the one hand, previous studies exhibit a high degree of methodological heterogeneity: the host species used (e.g., guinea pigs, rabbits, rats), injury models (e.g., transection and anastomosis, long-segment defects, clamp-compression injury), and intervention strategies (single PRP preparation versus PRP combined with specific stem cells or biomaterials) vary widely, making it difficult to objectively evaluate and compare the relative therapeutic contributions of individual mechanisms. On the other hand, the current positive therapeutic effects rely heavily on models of immediate repair following acute injury in young, healthy animals, which is significantly disconnected from clinical reality. In real-world clinical settings, patients with facial nerve palsy often present with systemic comorbidities such as diabetes and microcirculatory disorders, and many face delayed treatment and chronic neuromuscular atrophy due to missing the optimal window for surgery. Therefore, future investigations should prioritize chronic degeneration models and clinically relevant comorbidity models, including diabetes-associated nerve dysfunction and impaired microvascular conditions. Further clinical studies are required to determine standardized PRP preparation protocols, optimal dosage, delivery strategies, and treatment windows for facial nerve repair.

#### 4.2.3 Applications of PRP in the repair of other peripheral nerve injuries

Beyond the neurological conditions discussed in Section 5.2.1, PRP has also been investigated as a potential regenerative strategy for peripheral nerve injury, where modulation of axonal regeneration and the local repair microenvironment represents a major therapeutic challenge. In preclinical models, PRP administration has been reported to influence several aspects of peripheral nerve repair, including axonal growth, myelin preservation, Schwann cell activity, and functional recovery. However, the magnitude of these effects varies considerably depending on the injury model, nerve type, and evaluation criteria used. These potential regenerative effects have been described in diverse experimental settings, including cavernous nerve injury, age-associated nerve degeneration, and metabolically induced peripheral neuropathy (127–131). Although these findings suggest that PRP may influence common regenerative pathways, such as neurotrophic signaling and inflammatory modulation, whether these mechanisms are universally applicable across different nerve injuries remains uncertain. Nevertheless, the apparent consistency of these findings should be interpreted cautiously because most evidence originates from controlled animal experiments that may not fully reproduce the complexity of human peripheral nerve disorders.

Clinical evidence is more developed in carpal tunnel syndrome (CTS), which currently represents the most extensively studied peripheral nerve indication for PRP. Randomized and comparative clinical studies have generally reported reductions in pain severity and improvements in functional scores after PRP treatment for CTS. Some studies have also observed favorable electrophysiological changes; however, differences in injection protocols, PRP composition, comparator groups, and follow-up duration limit direct comparison among trials (132–136). Importantly, several studies also suggest that PRP may compare favorably with corticosteroid injection, indicating potential value as an alternative biologic intervention in selected CTS patients (133–135). This relative consistency distinguishes CTS from other peripheral nerve conditions, where clinical data remain far more limited.

Current evidence suggests that PRP has potential in peripheral nerve repair, with preclinical studies supporting a broad regenerative rationale and CTS providing the most substantial clinical signal to date. However, the field remains limited by marked heterogeneity in experimental design and treatment protocols. Further research should focus on clarifying whether PRP has indication-specific or broadly applicable neuroregenerative effects, while establishing standardized preparation and administration strategies to support more reliable clinical translation.

## 5 Discussion

**Figure 5:**
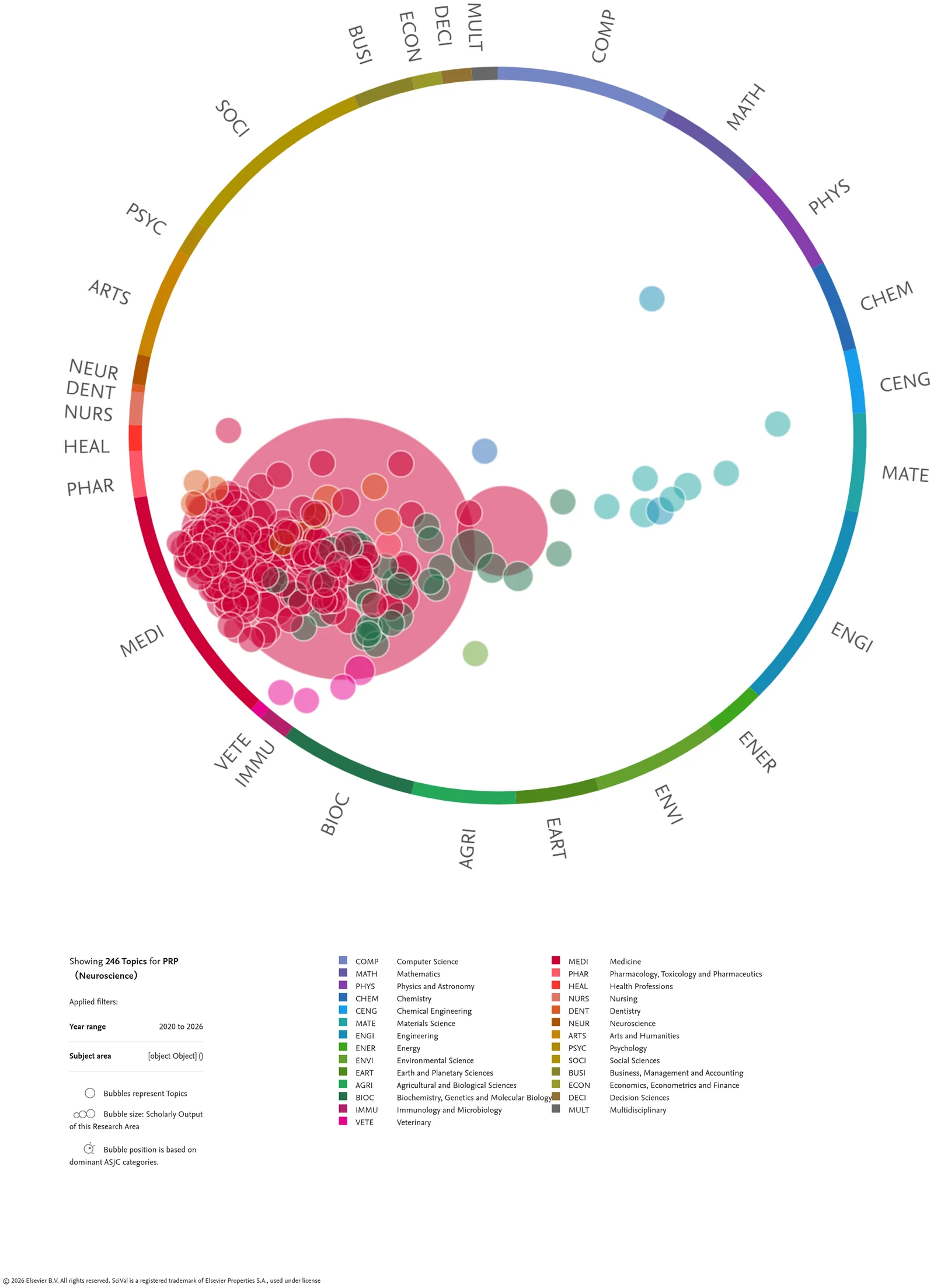
Multidisciplinary landscape and topical distribution of PRP research in neuroscience. Based on the All Science Journal Classification (ASJC) system, the topic wheel illustrates the cross-disciplinary integration of the field. The bubbles representing specific research topics are predominantly clustered in the lower-left quadrant. This spatial distribution demonstrates that the research spans from fundamental mechanisms in Biochemistry, Genetics, and Molecular Biology (BIOC) to core clinical applications in Medicine (MEDI), while closely aligning with Neuroscience (NEUR). Notably, the visible extension of topics toward Materials Science (MATE) visually confirms the emerging translational paradigm of combining PRP with advanced biomaterials for nerve conduit engineering. The figure was created using SciVal (Elsevier BV) using the “Topics” module. Bubble size denotes volume of publications, distance between bubbles reflects the semantic similarity of the dominant ASJC categories.

The multidisciplinary research landscape of PRP in neuroscience underscores its biological potential in peripheral nerve regeneration, neuropathic pain, and spinal cord repair (**Figure 5**). PRP and its derivatives may support neural repair by promoting angiogenesis, modulating inflammation, attenuating apoptosis, preserving cellular homeostasis, enhancing Schwann cell-associated regeneration, and regulating the immune microenvironment. The strongest rationale exists in peripheral nerve injury, where PRP facilitates axonal regrowth and remyelination, especially alongside stem cells, hydrogels, or nerve conduits. Conversely, central nervous system applications remain predominantly preclinical. Although pathways such as CXCL5/CXCR2 provide a mechanistic basis, clinical translation remains constrained by disease complexity and insufficient human evidence. Therefore, publication volume should not be equated with established efficacy. Insights must be drawn from the broader clinical trial landscape in regenerative medicine, where randomized controlled trials (RCTs) across musculoskeletal, cutaneous, and other indications demonstrate that biological plausibility does not consistently translate into clinical benefit.

Recent clinical studies indicate that the translation of PRP into routine practice remains constrained by several factors (7, 137, 138). Therapeutic efficacy is heterogeneous and, in some settings, difficult to distinguish from placebo, procedure-related effects, or natural healing. In addition, PRP may improve selected objective outcomes without producing parallel gains in patient-reported benefit, suggesting a dissociation between biological activity and clinically perceived improvement. Clinical interpretation is further complicated by the lack of product standardization, as autologous PRP varies substantially according to preparation protocols and donor-specific biological characteristics. Moreover, the small sample sizes of some prospective studies limit statistical power and reduce the generalizability of reported findings. Collectively, these issues indicate that PRP clinical translation is influenced by efficacy heterogeneity, placebo effects, endpoint dissociation, inadequate product standardization, and limited study scale.

Based on these considerations, the observed clinical heterogeneity should not be interpreted simply as evidence that PRP is ineffective as a whole, but rather as a product–disease interaction shaped by indication, pathological microenvironment, preparation method, and cellular composition. These factors also inform PRP subtype selection in neuromedicine. Experience from musculoskeletal disorders indicates that leukocyte content can influence clinical response, but the superiority of leukocyte-poor PRP versus leukocyte-rich PRP is indication-dependent rather than universal (139). In orthopedic settings, leukocyte-poor PRP has often been preferred when excessive inflammation may be detrimental, whereas leukocyte-rich PRP may retain stronger angiogenic, immunogenic and monocyte/macrophage recruiting effects. Recent data also suggest that leukocyte-rich PRP is not uniformly more pro-inflammatory than previously assumed (140, 141). This distinction is likely to be equally relevant in neurological disorders, where different pathological settings may require different inflammatory profiles. Leukocyte-poor PRP may be more appropriate when minimizing secondary neuroinflammation is a priority, whereas leukocyte-rich PRP may be advantageous when immune-cell-mediated debris clearance, vascular support, or antimicrobial protection is required. Based on this, PRP subtype selection represents only one dimension of clinical heterogeneity. Further analysis indicates that even within the same PRP subtype, the absolute platelet and leukocyte dose, as well as the degree of enrichment, may determine both the direction and magnitude of the therapeutic response.

Beyond subtype selection, another unresolved issue is the therapeutic threshold of PRP. Evidence from musculoskeletal research indicates that PRP efficacy is threshold-dependent rather than linearly dose-dependent, with outcomes shaped by the absolute platelet dose, the degree of enrichment above baseline, and the biological context of the target tissue. In clinical studies of knee osteoarthritis, an intra-articular dose of approximately 10 billion platelets per injection has been proposed as a meaningful threshold associated with more durable symptom improvement and modulation of inflammatory mediators, whereas lower doses may yield less consistent long-term benefit. At the same time, *in vitro* and tissue-repair studies suggest a biphasic response, with optimal biological activity often observed at platelet concentrations around 0.5 × 10^6^ to 1.0 × 10^6^/μL, while substantially higher concentrations may become inhibitory (**Table 2**), Although no universal threshold can yet be defined across all PRP formulations, derivatives, and indications, these findings suggest that neurological applications will also require indication-specific dosing windows rather than simple escalation of platelet concentration. Future progress in the field will therefore depend on rigorous product characterization, clearer reporting of platelet and leukocyte dose, indication-specific formulation selection, optimized delivery systems, and larger comparative studies directly evaluating PRP subtypes and dosing strategies in neurological disorders.

**Table 2:** Comparative analysis of representative studies addressing PRP efficacy threshold, platelet concentration/dose, and indication-specific interpretation.

Study (authors, year)	Indication/cell type	Platelet concentration/absolute dose tested	Key threshold/dose findings	Indication-specific interpretation & conclusion
Bansal, H.et al. (2021) (142)	Knee Osteoarthritis (OA)	PRP (~10 billion absolute platelets per dose) vs. Hyaluronic Acid (HA)	A minimum total absolute threshold of 10 billion (10×109) platelets is crucial for long-term clinical efficacy and chondroprotection.	Absolute platelet count per injection, rather than volume or concentration ratio alone, is critical for sustained clinical improvement (WOMAC/IKDC) and anti-inflammatory action up to 1 year in knee OA.
Giusti, I.et al. (2014) (143)	Human Tenocytes	Range up to 5.0×10^6^/μL	Biphasic/Bell-shaped response: Intermediate concentrations (0.5×10^6^ and 1.0×10^6^/μL) optimal for proliferation/collagen synthesis. High concentrations (≥3.0–5.0×10^6^/μL) inhibited proliferation, decreased collagen, and up-regulated MMPs.	Excessively high PRP concentrations are counterproductive for tendon healing; a balanced intermediate concentration is required to avoid tissue degradation by matrix metalloproteinases (MMPs).
Jo, C. H.et al. (2012) (144)	Rotator Cuff Tenocytes	Range: 1.0×10^5^to 1.6×107/μL	Cell proliferation peaked at 0.5–1.0×10^6^/μL (500–1000×10^3^/μL). Higher doses showed a plateau or diminished marginal benefit.	Degenerative rotator cuff tenocytes require an optimal concentration range for collagen/GAG matrix gene expression, beyond which higher concentrations offer no added benefit.
Han, J.et al. (2007) (145)	Human Periodontal Ligament Cells (hPDLCs)	Various PRP concentrations (activated vs. non-activated)	Low to moderate PRP concentrations promoted proliferation and ALP activity, whereas high concentrations inhibited cell proliferation.	Periodontal tissue regeneration requires strict control over PRP dose, as over-concentrated PRP suppresses PDLC growth and differentiation.
Slapnicka, J.et al.(2008) (146)	Osteoblasts and Fibroblasts	Range from 0.38× to 0.95× baseline increase up to high concentrations	Maximal stimulatory effect observed at around 1.5×10^6^/μL (10% PRP concentration). Higher concentrations showed no further stimulation or inhibitory effects.	Bone and hard tissue engineering displays a clear therapeutic window; excessive platelet counts do not correlate with proportional osteoblast proliferation.
Mishra, A.et al. (2009) (147)	Mesenchymal Stem Cells (MSCs)	Inactivated, buffered PRP vs. Control	Buffered PRP significantly enhanced MSC proliferation (1.041 vs. 0.199, p<0.001) and upregulated chondrogenic gene markers (Sox-9, Aggrecan).	Neutralizing/buffering PRP pH and maintaining appropriate growth factor levels is key to stimulating stem cell expansion and cartilage matrix differentiation.
Berger, D. R.et al. (2019) (148)	Human Tenocytes (Young vs. Aged Donors)	Platelet Lysates (PL) across concentration gradients	Dose-dependent increase in tenocyte proliferation/migration across concentrations, with age of donors modulating specific protein release profiles.	PRP/PL efficacy is influenced both by total platelet protein concentration and donor age, highlighting the need for donor-tailored dosing strategies.

## References

[1] Mormone E D'Esposito V De Luca P Ferrara FEO Bellotti FP Formisano P . Platelet-rich plasma from the research to the clinical arena: A journey toward the precision regenerative medicine. Int J Mol Sci. (2026) 27(2):1058. doi: 10.3390/ijms27021058 41596701 PMC12842059

[2] Boswell SG Cole BJ Sundman EA Karas V Fortier LA . Platelet-rich plasma: A milieu of bioactive factors. Arthroscopy. (2012) 28:429–39. doi: 10.1016/j.arthro.2011.10.018 22284405

[3] Sundman EA Cole BJ Fortier LA . Growth factor and catabolic cytokine concentrations are influenced by the cellular composition of platelet-rich plasma. Am J Sports Med. (2011) 39:2135–40. doi: 10.1177/0363546511417792 21846925

[4] Pidlisetsky АТ Kosiakova GV Goridko TM Berdyschev AG Meged OF Savosko SI . Administration of platelet-rich plasma or concentrated bone marrow aspirate after mechanically induced ischemia improves biochemical parameters in skeletal muscle. Ukr Biochem J. (2021) 93:30–8. doi: 10.15407/ubj93.03.030

[5] Olifirenko O Savosko S Movchan O . Knee joint structural changes in osteoarthritis and injections of platelet rich plasma and bone marrow aspirate concentrate. Georgian Med News. (2020) (303):184–8. 32841203

[6] Bennell KL Paterson KL Metcalf BR Duong V Eyles J Kasza J . Effect of intra-articular platelet-rich plasma vs placebo injection on pain and medial tibial cartilage volume in patients with knee osteoarthritis: The restore randomized clinical trial. Jama. (2021) 326:2021–30. doi: 10.1001/jama.2021.19415 34812863 PMC8611484

[7] Paget LDA Reurink G de Vos RJ Weir A Moen MH Bierma-Zeinstra SMA . Platelet-rich plasma injections for the treatment of ankle osteoarthritis. Am J Sports Med. (2023) 51:2625–34. doi: 10.1177/03635465231182438 37417359 PMC10394962

[8] Raeissadat SA Ghazi Hosseini P Bahrami MH Salman Roghani R Fathi M Gharooee Ahangar A . The comparison effects of intra-articular injection of platelet rich plasma (Prp), plasma rich in growth factor (Prgf), hyaluronic acid (Ha), and ozone in knee osteoarthritis; a one year randomized clinical trial. BMC Musculoskelet Disord. (2021) 22:134. doi: 10.1186/s12891-021-04017-x 33536010 PMC7860007

[9] Saumell-Esnaola M Delgado D García Del Caño G Beitia M Sallés J González-Burguera I . Isolation of platelet-derived exosomes from human platelet-rich plasma: Biochemical and morphological characterization. Int J Mol Sci. (2022) 23(5):2861. doi: 10.3390/ijms23052861 35270001 PMC8911307

[10] Anitua E Troya M Falcon-Pérez JM López-Sarrio S González E Alkhraisat MH . Advances in platelet rich plasma-derived extracellular vesicles for regenerative medicine: A systematic-narrative review. Int J Mol Sci. (2023) 24. doi: 10.3390/ijms241713043 37685849 PMC10488108

[11] Wu WS Chen LR Chen KH . Platelet-rich plasma (Prp): Molecular mechanisms, actions and clinical applications in human body. Int J Mol Sci. (2025) 26. doi: 10.3390/ijms262110804 41226837 PMC12608683

[12] Gupta AK Wang T Rapaport JA Talukder M . Therapeutic potential of extracellular vesicles (Exosomes) derived from platelet-rich plasma: A literature review. J Cosmet Dermatol. (2025) 24:e16709. doi: 10.1111/jocd.16709 39618056 PMC11845942

[13] Rak J . Microparticles in cancer. Semin Thromb Hemost. (2010) 36:888–906. doi: 10.1055/s-0030-1267043 21049390

[14] Vidal-Negreira F García-González M Valiño-Cultelli V González-Cantalapiedra A . Platelet-rich plasma in veterinary orthopedic surgery: A systematic review and quality evaluation on liquid- and gel-based therapies in dogs. Gels. (2025) 11(12):994. doi: 10.3390/gels11120994 41441150 PMC12732500

[15] Wang SL Liu XL Kang ZC Wang YS . Platelet-rich plasma promotes peripheral nerve regeneration after sciatic nerve injury. Neural Regener Res. (2023) 18:375–81. doi: 10.4103/1673-5374.346461 35900433 PMC9396478

[16] Zhang Y Yi D Hong Q Cao J Geng X Liu J . Platelet-rich plasma-derived exosomes boost mesenchymal stem cells to promote peripheral nerve regeneration. J Control Release. (2024) 367:265–82. doi: 10.1016/j.jconrel.2024.01.043 38253204

[17] Shang K Liu Y Qadeer A . Platelet-rich plasma in peripheral nerve injury repair: A comprehensive review of mechanisms, clinical applications, and therapeutic potential. Exp Biol Med (Maywood). (2025) 250:10746. doi: 10.3389/ebm.2025.10746 41063785 PMC12500476

[18] Chen NF Sung CS Wen ZH Chen CH Feng CW Hung HC . Therapeutic effect of platelet-rich plasma in rat spinal cord injuries. Front Neurosci. (2018) 12:252. doi: 10.3389/fnins.2018.00252 29740270 PMC5924817

[19] Anitua E Pascual C Antequera D Bolos M Padilla S Orive G . Plasma rich in growth factors (Prgf-Endoret) reduces neuropathologic hallmarks and improves cognitive functions in an Alzheimer's disease mouse model. Neurobiol Aging. (2014) 35:1582–95. doi: 10.1016/j.neurobiolaging.2014.01.009 24524966

[20] Kong H Luo J Zou Z Li Y Tang X Han J . Exercise-derived exosomes: Molecular mediators of systemic health and disease therapy. J Nanobiotechnology. (2026) 24(1):232. doi: 10.1186/s12951-026-04115-9 41654894 PMC12977883

[21] Dohan Ehrenfest DM Rasmusson L Albrektsson T . Classification of platelet concentrates: From pure platelet-rich plasma (P-Prp) to leucocyte- and platelet-rich fibrin (L-Prf). Trends Biotechnol. (2009) 27:158–67. doi: 10.1016/j.tibtech.2008.11.009 19187989

[22] DeLong JM Russell RP Mazzocca AD . Platelet-rich plasma: The paw classification system. Arthroscopy. (2012) 28:998–1009. doi: 10.1016/j.arthro.2012.04.148 22738751

[23] Mishra A Harmon K Woodall J Vieira A . Sports medicine applications of platelet rich plasma. Curr Pharm Bio/Technol. (2012) 13:1185–95. doi: 10.2174/138920112800624283 21740373

[24] Mautner K Malanga GA Smith J Shiple B Ibrahim V Sampson S . A call for a standard classification system for future biologic research: The rationale for new Prp nomenclature. Pm R. (2015) 7:S53–9. doi: 10.1016/j.pmrj.2015.02.005 25864661

[25] Magalon J Chateau AL Bertrand B Louis ML Silvestre A Giraudo L . Depa classification: A proposal for standardising Prp use and a retrospective application of available devices. BMJ Open Sport Exerc Med. (2016) 2:e000060. doi: 10.1136/bmjsem-2015-000060 27900152 PMC5117023

[26] Lana J Purita J Paulus C Huber SC Rodrigues BL Rodrigues AA . Contributions for classification of platelet rich plasma - Proposal of a new classification: Marspill. Regener Med. (2017) 12:565–74. doi: 10.2217/rme-2017-0042 28758836

[27] Harrison P . The use of platelets in regenerative medicine and proposal for a new classification system: Guidance from the Ssc of the Isth. J Thromb Haemost. (2018) 16:1895–900. doi: 10.1111/jth.14223 30099839

[28] Acebes-Huerta A Arias-Fernández T Bernardo Á Muñoz-Turrillas MC Fernández-Fuertes J Seghatchian J . Platelet-derived bio-products: Classification update, applications, concerns and new perspectives. Transfus Apher Sci. (2020) 59:102716. doi: 10.1016/j.transci.2019.102716 31928859

[29] Kon E Di Matteo B Delgado D Cole BJ Dorotei A Dragoo JL . Platelet-rich plasma for the treatment of knee osteoarthritis: An expert opinion and proposal for a novel classification and coding system. Expert Opin Biol Ther. (2020) 20:1447–60. doi: 10.1080/14712598.2020.1798925 32692595

[30] Piao L Park H Jo CH . Theoretical prediction and validation of cell recovery rates in preparing platelet-rich plasma through a centrifugation. PLoS One. (2017) 12:e0187509. doi: 10.1371/journal.pone.0187509 29095890 PMC5667898

[31] Amable PR Carias RB Teixeira MV da Cruz Pacheco I Corrêa do Amaral RJ Granjeiro JM . Platelet-rich plasma preparation for regenerative medicine: Optimization and quantification of cytokines and growth factors. Stem Cell Res Ther. (2013) 4:67. doi: 10.1186/scrt218 23759113 PMC3706762

[32] Araki J Jona M Eto H Aoi N Kato H Suga H . Optimized preparation method of platelet-concentrated plasma and noncoagulating platelet-derived factor concentrates: Maximization of platelet concentration and removal of fibrinogen. Tissue Eng Part C Methods. (2012) 18:176–85. doi: 10.1089/ten.TEC.2011.0308 21951067 PMC3285602

[33] Magalon J Brandin T Francois P Degioanni C De Maria L Grimaud F . Technical and biological review of authorized medical devices for platelets-rich plasma preparation in the field of regenerative medicine. Platelets. (2021) 32:200–8. doi: 10.1080/09537104.2020.1832653 33155867

[34] Fadadu PP Mazzola AJ Hunter CW Davis TT . Review of concentration yields in commercially available platelet-rich plasma (Prp) systems: A call for Prp standardization. Reg Anesth Pain Med. (2019). doi: 10.1136/rapm-2018-100356 30992411

[35] Zimmermann R Arnold D Strasser E Ringwald J Schlegel A Wiltfang J . Sample preparation technique and white cell content influence the detectable levels of growth factors in platelet concentrates. Vox Sang. (2003) 85:283–9. doi: 10.1111/j.0042-9007.2003.00361.x 14633254

[36] Li M Zhao Y Chen X Du X Luo Y Li Y . Comparative analysis of the quality of platelet concentrates produced by apheresis procedures, platelet rich plasma, and buffy coat. Transfusion. (2024) 64:367–79. doi: 10.1111/trf.17704 38174435

[37] Noulsri E Udomwinijsilp P Lerdwana S Chongkolwatana V Permpikul P . Differences in levels of platelet-derived microparticles in platelet components prepared using the platelet rich plasma, buffy coat, and apheresis procedures. Transfus Apher Sci. (2017) 56:135–40. doi: 10.1016/j.transci.2016.10.006 28029568

[38] Krüger JP Hondke S Endres M Pruss A Siclari A Kaps C . Human platelet-rich plasma stimulates migration and chondrogenic differentiation of human subchondral progenitor cells. J Orthop Res. (2012) 30:845–52. doi: 10.1002/jor.22005 22058056

[39] Miron RJ Pikos MA Estrin NE Kobayashi-Fujioka M Espinoza AR Basma H . Extended platelet-rich fibrin. Periodontol 2000. (2024) 94:114–30. doi: 10.1111/prd.12537 37986559

[40] Miron RJ Gruber R Farshidfar N Sculean A Zhang Y . Ten years of injectable platelet-rich fibrin. Periodontol 2000. (2024) 94:92–113. doi: 10.1111/prd.12538 38037213

[41] Miron RJ Fujioka-Kobayashi M Sculean A Zhang Y . Optimization of platelet-rich fibrin. Periodontol 2000. (2024) 94:79–91. doi: 10.1111/prd.12521 37681522

[42] Yun Z Wu J Sun X Yu T Xue W Dai A . Neural-enhancing Prp/Alg/Gelma triple-network hydrogel for neurogenesis and angiogenesis after spinal cord injury via Pi3k/Akt/Mtor signaling pathway. Theranostics. (2025) 15:3837–61. doi: 10.7150/thno.109091 40213674 PMC11980671

[43] Kim JY Jeon WJ Kim DH Rhyu IJ Kim YH Youn I . An inside-out vein graft filled with platelet-rich plasma for repair of a short sciatic nerve defect in rats. Neural Regener Res. (2014) 9:1351–7. doi: 10.4103/1673-5374.137587 25221591 PMC4160865

[44] Dong Q Yang X Liang X Liu J Wang B Zhao Y . Composite hydrogel conduit incorporated with platelet-rich plasma improved the regenerative microenvironment for peripheral nerve repair. ACS Appl Mater Interfaces. (2023) 15:24120–33. doi: 10.1021/acsami.3c02548 37162458

[45] Teymur H Tiftikcioglu YO Cavusoglu T Tiftikcioglu BI Erbas O Yigitturk G . Effect of platelet-rich plasma on reconstruction with nerve autografts. Kaohsiung J Med Sci. (2017) 33:69–77. doi: 10.1016/j.kjms.2016.11.005 28137414 PMC11916268

[46] Lichtenfels M Colomé L Sebben AD Braga-Silva J . Effect of platelet rich plasma and platelet rich fibrin on sciatic nerve regeneration in a rat model. Microsurgery. (2013) 33:383–90. doi: 10.1002/micr.22105 23640879

[47] Yuan B Zheng X Wu ML Yang Y Chen JW Gao HC . Platelet-rich plasma gel-loaded collagen/chitosan composite film accelerated rat sciatic nerve injury repair. ACS Omega. (2023) 8:2931–41. doi: 10.1021/acsomega.2c05351 36713745 PMC9878625

[48] Zhang Y Yi D Hong Q Liu C Chi K Liu J . Platelet-rich plasma-derived exosomes enhance mesenchymal stem cell paracrine function and nerve regeneration potential. Biochem Biophys Res Commun. (2024) 699:149496. doi: 10.1016/j.bbrc.2024.149496 38290175

[49] Sánchez M Anitua E Delgado D Sanchez P Prado R Orive G . Platelet-rich plasma, a source of autologous growth factors and biomimetic scaffold for peripheral nerve regeneration. Expert Opin Biol Ther. (2017) 17:197–212. doi: 10.1080/14712598.2017.1259409 27845852

[50] Sánchez M Yoshioka T Ortega M Delgado D Anitua E . Ultrasound-guided platelet-rich plasma injections for the treatment of common peroneal nerve palsy associated with multiple ligament injuries of the knee. Knee Surg Sports Traumatol Arthrosc. (2014) 22:1084–9. doi: 10.1007/s00167-013-2479-y 23519544

[51] Bohren Y Timbolschi DI Muller A Barrot M Yalcin I Salvat E . Platelet-rich plasma and cytokines in neuropathic pain: A narrative review and a clinical perspective. Eur J Pain. (2022) 26:43–60. doi: 10.1002/ejp.1846 34288258

[52] Bendinelli P Matteucci E Dogliotti G Corsi MM Banfi G Maroni P . Molecular basis of anti-inflammatory action of platelet-rich plasma on human chondrocytes: Mechanisms of nf-κb inhibition via hgf. J Cell Physiol. (2010) 225:757–66. doi: 10.1002/jcp.22274 20568106

[53] Matsui M Tabata Y . Enhanced angiogenesis by multiple release of platelet-rich plasma contents and basic fibroblast growth factor from gelatin hydrogels. Acta Biomater. (2012) 8:1792–801. doi: 10.1016/j.actbio.2012.01.016 22293581

[54] Anitua E Pascual C Pérez-Gonzalez R Antequera D Padilla S Orive G . Intranasal delivery of plasma and platelet growth factors using prgf-endoret system enhances neurogenesis in a mouse model of Alzheimer's disease. PloS One. (2013) 8:e73118. doi: 10.1371/journal.pone.0073118 24069173 PMC3777974

[55] Everts P Onishi K Jayaram P Lana JF Mautner K . Platelet-rich plasma: New performance understandings and therapeutic considerations in 2020. Int J Mol Sci. (2020) 21. doi: 10.3390/ijms21207794 33096812 PMC7589810

[56] Sheean AJ Anz AW Bradley JP . Platelet-rich plasma: Fundamentals and clinical applications. Arthroscopy. (2021) 37:2732–4. doi: 10.1016/j.arthro.2021.07.003 34481615

[57] Cieslik-Bielecka A Dohan Ehrenfest DM Lubkowska A Bielecki T . Microbicidal properties of leukocyte- and platelet-rich plasma/fibrin (l-prp/l-prf): New perspectives. J Biol Regul Homeost Agents. (2012) 26:43s–52s. 23648198

[58] Guo S Wang H Yin Y . Microglia polarization from m1 to m2 in neurodegenerative diseases. Front Aging Neurosci. (2022) 14:815347. doi: 10.3389/fnagi.2022.815347 35250543 PMC8888930

[59] Liddelow SA Guttenplan KA Clarke LE Bennett FC Bohlen CJ Schirmer L . Neurotoxic reactive astrocytes are induced by activated microglia. Nature. (2017) 541:481–7. doi: 10.1038/nature21029 28099414 PMC5404890

[60] Xu H Lotfy P Gelb S Pragana A Hehnly C Byer LIJ . The choroid plexus synergizes with immune cells during neuroinflammation. Cell. (2024) 187:4946–63.e17. doi: 10.1016/j.cell.2024.07.002 39089253 PMC11458255

[61] Robert SM Reeves BC Kiziltug E Duy PQ Karimy JK Mansuri MS . The choroid plexus links innate immunity to csf dysregulation in hydrocephalus. Cell. (2023) 186:764–85.e21. doi: 10.1016/j.cell.2023.01.017 36803604 PMC10069664

[62] Islam J Chaudhary PK Kim S Kc E Park YS Kim S . The efficacy of intrathecal platelet-rich plasma administration in alleviation of chronic neuropathic pain in rat model. Mol Neurobiol. (2025) 62:13840–57. doi: 10.1007/s12035-025-05173-0 40601220 PMC12511181

[63] Sheean AJ Anz AW Bradley JP Chu HI Wang JW Wood SH . Intrathecal autologous thrombin-activated condensed platelet cytokines in chronic neurodegenerative disease: A hypothesis for anti-inflammatory and regenerative response. Neuro Endocrinol Lett. (2023) 44:418–25. 37776559

[64] Bukhari JI Alghamdi AE Albeladi SM Danish HE Alqarni RB Almutairi DM . Efficacy of platelet-rich plasma in endoscopic sinus surgery for chronic sinusitis: A systematic review and meta-analysis. Cureus. (2024) 16:e76568. doi: 10.7759/cureus.76568 39877770 PMC11774301

[65] NyamErdene A Le NTN Nebie O Faivre E Delila L Chou ML . Platelet concentrate-derived extracellular vesicles promote adult hippocampal neurogenesis. Biomaterials. (2026) 328:123838. doi: 10.1016/j.biomaterials.2025.123838 41218272

[66] Coucke B Van Hoylandt A van Loon J Van Calenbergh F Van Gerven L Theys T . Leukocyte- and platelet-rich fibrin in cranial surgery: Study protocol for a prospective, parallel-group, single-blinded randomized controlled non-inferiority trial {1}. Trials. (2023) 24:219. doi: 10.1186/s13063-023-07252-w 36959672 PMC10034240

[67] Gunaydin B Acar M Emmez G Akcali D Tokgoz N . Epidural patch with autologous platelet rich plasma: A novel approach. J Anesth. (2017) 31:907–10. doi: 10.1007/s00540-017-2400-9 28823090

[68] Guler S Akcali O Sen B Micili SC Sanli NK Cankaya D . Effect of platelet-rich plasma, fat pad and dural matrix in preventing epidural fibrosis. Acta Ortop Bras. (2020) 28:31–5. doi: 10.1590/1413-785220202801218823 32095110 PMC7006529

[69] Sansone L Reali V Pellegrini L Villanova L Aventaggiato M Marfe G . Sirt1 silencing confers neuroprotection through igf-1 pathway activation. J Cell Physiol. (2013) 228:1754–61. doi: 10.1002/jcp.24334 23359486

[70] Lei Q Pan Q Li N Zhou Z Zhang J He X . H19 regulates the proliferation of bovine male germline stem cells via igf-1 signaling pathway. J Cell Physiol. (2018) 234:915–26. doi: 10.1002/jcp.26920 30069947

[71] Funa K Sasahara M . The roles of pdgf in development and during neurogenesis in the normal and diseased nervous system. J Neuroimmune Pharmacol. (2014) 9:168–81. doi: 10.1007/s11481-013-9479-z 23771592 PMC3955130

[72] Wang Y Li J . Current progress in growth factors and extracellular vesicles in tendon healing. Int Wound J. (2023) 20:3871–83. doi: 10.1111/iwj.14261 37291064 PMC10588330

[73] Zheng C Zhu Q Liu X Huang X He C Jiang L . Improved peripheral nerve regeneration using acellular nerve allografts loaded with platelet-rich plasma. Tissue Eng Part A. (2014) 20:3228–40. doi: 10.1089/ten.TEA.2013.0729 24901030 PMC4259182

[74] Ikumi A Hara Y Yoshioka T Kanamori A Yamazaki M . Effect of local administration of platelet-rich plasma (prp) on peripheral nerve regeneration: An experimental study in the rabbit model. Microsurgery. (2018) 38:300–9. doi: 10.1002/micr.30263 29094404

[75] Takeuchi M Kamei N Shinomiya R Sunagawa T Suzuki O Kamoda H . Human platelet-rich plasma promotes axon growth in brain-spinal cord coculture. Neuroreport. (2012) 23:712–6. doi: 10.1097/WNR.0b013e3283567196 22750774

[76] Tao X Liu K Li W Zhao S Liu C Dai Q . Saponin of aralia taibaiensis promotes angiogenesis through vegf/vegfr2 signaling pathway in cerebral ischemic mice. J Ethnopharmacol. (2023) 317:116771. doi: 10.1016/j.jep.2023.116771 37308026

[77] Cattin AL Burden JJ Van Emmenis L Mackenzie FE Hoving JJ Garcia Calavia N . Macrophage-induced blood vessels guide schwann cell-mediated regeneration of peripheral nerves. Cell. (2015) 162:1127–39. doi: 10.1016/j.cell.2015.07.021 26279190 PMC4553238

[78] She S Ren L Chen P Wang M Chen D Wang Y . Functional roles of chemokine receptor ccr2 and its ligands in liver disease. Front Immunol. (2022) 13:812431. doi: 10.3389/fimmu.2022.812431 35281057 PMC8913720

[79] Yadav S Srivastava S Singh G . Platelet-rich plasma exhibits anti-inflammatory effect and attenuates cardiomyocyte damage by reducing nf-κb and enhancing vegf expression in isoproterenol induced cardiotoxicity model. Environ Toxicol. (2022) 37:936–53. doi: 10.1002/tox.23456 35014750

[80] Akbari-Gharalari N Ghahremani-Nasab M Naderi R Aliyari-Serej Z Karimipour M Shahabi P . Improvement of spinal cord injury symptoms by targeting the bax/bcl2 pathway and modulating tnf-α/il-10 using platelet-rich plasma exosomes loaded with dexamethasone. AIMS Neurosci. (2023) 10:332–53. doi: 10.3934/Neuroscience.2023026 38188010 PMC10767060

[81] Akbari-Gharalari N Aliyari-Serej Z Ghahremani-Nasab M Zangbar HS Yahyavi Y Nezhadshahmohammad F . Cerebrolysin-loaded platelet-rich plasma exosomes: Restoring immune homeostasis via tnf-α/il-10 modulation and apoptosis targeting for spinal cord injury repair. J Spinal Cord Med. (2026) 49:375–87. doi: 10.1080/10790268.2025.2503053 40470992 PMC12931343

[82] Wu J Piao Y Liu Q Yang X . Platelet-rich plasma-derived extracellular vesicles: A superior alternative in regenerative medicine? Cell Prolif. (2021) 54:e13123. doi: 10.1111/cpr.13123 34609779 PMC8666280

[83] Berndt S Carpentier G Turzi A Borlat F Cuendet M Modarressi A . Angiogenesis is differentially modulated by platelet-derived products. Biomedicines. (2021) 9(3):251. doi: 10.3390/biomedicines9030251 33806471 PMC8000116

[84] He L Zhao N Chen X Zhang W Lv K Xu Y . Platelet-rich plasma-derived exosomes accelerate the healing of diabetic foot ulcers by promoting macrophage polarization toward the m2 phenotype. Clin Exp Med. (2025) 25:163. doi: 10.1007/s10238-025-01651-w 40372505 PMC12081558

[85] Chen C Xu HH Liu XY Zhang YS Zhong L Wang YW . 3d printed collagen/silk fibroin scaffolds carrying the secretome of human umbilical mesenchymal stem cells ameliorated neurological dysfunction after spinal cord injury in rats. Regener Biomater. (2022) 9:rbac014. doi: 10.1093/rb/rbac014 35480857 PMC9036898

[86] Chaudhari LR Kawale AA Sonkawade O Damle M Patil J Desai S . Activated platelet-rich plasma fibrin scaffolds enhance axonal regeneration and functional recovery following spinal cord injury. Ann BioMed Eng. (2026) 54:813–32. doi: 10.1007/s10439-025-03922-9 41324736

[87] Li L Zhang Y Mu J Chen J Zhang C Cao H . Transplantation of human mesenchymal stem-cell-derived exosomes immobilized in an adhesive hydrogel for effective treatment of spinal cord injury. Nano Lett. (2020) 20:4298–305. doi: 10.1021/acs.nanolett.0c00929 32379461

[88] Sun D Yu H Shen H Wang Y Su T Guo H . Sodium alginate hydrogel platelet rich plasma exosome loaded for synergistic hemostasis. Int J Biol Macromol. (2025) 311:143447. doi: 10.1016/j.ijbiomac.2025.143447 40306503

[89] Mao D Chen Q Tong S Xu Z Yu G Chang C . Integrated bioinformatics analysis identified cuproptosis-related hub gene mpeg1 as potential biomarker in spinal cord injury. Sci Rep. (2025) 15:1993. doi: 10.1038/s41598-025-86170-0 39814871 PMC11736097

[90] Cheng T Cao Y Liang B Yu K . Nanomaterial-based therapeutic strategies for spinal cord injury repair: Harnessing multifunctionality to overcome pathophysiological challenges. Biomater Sci. (2026) 14:393–425. doi: 10.1039/d5bm01238d 41379090

[91] Salarinia R Hosseini M Mohamadi Y Ghorbani A Alamdari DH Mafinezhad A . Combined use of platelet-rich plasma and adipose tissue-derived mesenchymal stem cells shows a synergistic effect in experimental spinal cord injury. J Chem Neuroanat. (2020) 110:101870. doi: 10.1016/j.jchemneu.2020.101870 33038437

[92] Hu ZB Chen HC Wei B Zhang ZM Wu SK Sun JC . Platelet rich plasma enhanced neuro-regeneration of human dental pulp stem cells *in vitro* and in rat spinal cord. Ann Transl Med. (2022) 10:584. doi: 10.21037/atm-22-1745 35722381 PMC9201165

[93] Zhao T Yan W Xu K Qi Y Dai X Shi Z . Combined treatment with platelet-rich plasma and brain-derived neurotrophic factor-overexpressing bone marrow stromal cells supports axonal remyelination in a rat spinal cord hemi-section model. Cytotherapy. (2013) 15:792–804. doi: 10.1016/j.jcyt.2013.04.004 23731762

[94] Botella Lucena P Heneka MT . Inflammatory aspects of Alzheimer's disease. Acta Neuropathol. (2024) 148:31. doi: 10.1007/s00401-024-02790-2 39196440

[95] Mattsson-Carlgren N Janelidze S Palmqvist S Cullen N Svenningsson AL Strandberg O . Longitudinal plasma p-tau217 is increased in early stages of Alzheimer's disease. Brain. (2020) 143:3234–41. doi: 10.1093/brain/awaa286 33068398 PMC7719022

[96] Vismara M Trivigno SMG Zarà M Momi S Gresele P Camera M . Platelet hyperreactivity and frailty in a mouse model of Alzheimer's disease are prevented by anti-oxidant treatment. Geroscience. (2026) 48:879–96. doi: 10.1007/s11357-025-01710-w 40459816 PMC12972370

[97] Trostchansky A Alarcón M . Platelet cox and lox enzymes orchestrate amyloid-β secretion via rhoa signaling: Implications for neurodegenerative diseases. Free Radic Biol Med. (2025) 240:641–9. doi: 10.1016/j.freeradbiomed.2025.08.060 40907839

[98] Ji L Jin RJ Li L . Platelet-rich plasma improves radiotherapy-induced emotional disorder and cognitive dysfunction, neuroinflammation in aged rats by inhibiting the activation of nlrp3 inflammasomes. Neurochem Res. (2023) 48:2531–41. doi: 10.1007/s11064-023-03933-9 37043084

[99] Moon JH Choi A Noh HJ Song JH Hong GL Lee NS . Platelet-rich plasma protects hippocampal neurons and memory functions in a rat model of vascular dementia. Anat Cell Biol. (2024) 57:559–69. doi: 10.5115/acb.24.117 39164249 PMC11663515

[100] Firoozi A Alizadeh A Zarifkar A Esmaeilpour T Namavar MR Alavi O . Comparison of the efficacy of human umbilical cord mesenchymal stem cells conditioned medium and platelet-rich plasma on the hippocampus of stz-induced rat model of Alzheimer's disease: A behavioral and stereological study. IBRO Neurosci Rep. (2023) 15:209–17. doi: 10.1016/j.ibneur.2023.09.006 37780033 PMC10539893

[101] Le NTN Han CL Delila L Nebie O Chien HT Wu YW . Proteomics of human platelet lysates and insight from animal studies on platelet protein diffusion to hippocampus upon intranasal administration. APL Bioeng. (2024) 8:026111. doi: 10.1063/5.0196553 38726021 PMC11080963

[102] Delila L Nebie O Le NTN Timmerman K Lee DY Wu YW . Neuroprotective effects of intranasal extracellular vesicles from human platelet concentrates supernatants in traumatic brain injury and Parkinson's disease models. J BioMed Sci. (2024) 31:87. doi: 10.1186/s12929-024-01072-z 39237980 PMC11375990

[103] de Rezende VL de Aguiar da Costa M Martins CD Mathias K Gonçalves CL Barichello T . Systemic rejuvenating interventions: Perspectives on neuroinflammation and blood-brain barrier integrity. Neurochem Res. (2025) 50:112. doi: 10.1007/s11064-025-04361-7 40035979

[104] Liu P Li H Wang Y Su X Li Y Yan M . Harmine ameliorates cognitive impairment by inhibiting Nlrp3 inflammasome activation and enhancing the Bdnf/Trkb signaling pathway in Stz-induced diabetic rats. Front Pharmacol. (2020) 11:535. doi: 10.3389/fphar.2020.00535 32425784 PMC7206617

[105] Chiasseu M Fesharaki-Zadeh A Saito T Saido TC Strittmatter SM . Gene-environment interaction promotes Alzheimer's risk as revealed by synergy of repeated mild traumatic brain injury and mouse App knock-in. Neurobiol Dis. (2020) 145:105059. doi: 10.1016/j.nbd.2020.105059 32858147 PMC7572902

[106] Panayi N Schulz P He P Hanna B Lifshitz J Rowe RK . Traumatic brain injury in mice generates early-stage Alzheimer's disease related protein pathology that correlates with neurobehavioral deficits. Mol Neurobiol. (2024) 61:7567–82. doi: 10.1007/s12035-024-04035-5 38411868 PMC11415463

[107] Cheng Z Wang H Geng X Rajah GB Elmadhoun O Peng G . Time and tissue windows in futile reperfusion after ischemic stroke. Aging Dis. (2024) 16:2544–52. doi: 10.14336/ad.2024.1024 39500359 PMC12339089

[108] Sun MS Jin H Sun X Huang S Zhang FL Guo ZN . Free radical damage in ischemia-reperfusion injury: An obstacle in acute ischemic stroke after revascularization therapy. Oxid Med Cell Longev. (2018) 2018:3804979. doi: 10.1155/2018/3804979 29770166 PMC5892600

[109] Miyauchi Y Miyamoto N Inaba T Xu HB Kijima C Hira K . Exercise-induced extracellular vesicles derived from platelet-rich plasma improved recovery after ischemic stroke. J Cereb Blood Flow Metab. (2026) 46:359–76. doi: 10.1177/0271678x251369219 40832971 PMC12901836

[110] Yang LT Li LH Peng WN . Platelet rich plasma alleviates Ogd/R injury in N2a cell by enhancing autophagy through the Mir-223/Paqr3 pathway. Acta Neurobiol Exp (Wars). (2022) 82:121–32. doi: 10.55782/ane-2022-011 35833812

[111] Zhang Y Ying G Ren C Jizhang Y Brogan D Liu Z . Administration of human platelet-rich plasma reduces infarction volume and improves motor function in adult rats with focal ischemic stroke. Brain Res. (2015) 1594:267–73. doi: 10.1016/j.brainres.2014.10.035 25452023

[112] Yip HK Chen KH Dubey NK Sun CK Deng YH Su CW . Cerebro- and renoprotective activities through platelet-derived biomaterials against cerebrorenal syndrome in rat model. Biomaterials. (2019) 214:119227. doi: 10.1016/j.biomaterials.2019.119227 31174067

[113] Dong X Yang Y Bao Z Midgley AC Li F Dai S . Micro-nanofiber composite biomimetic conduits promote long-gap peripheral nerve regeneration in canine models. Bioact Mater. (2023) 30:98–115. doi: 10.1016/j.bioactmat.2023.06.015 37560200 PMC10406865

[114] Zhu H Xue C Yao M Wang H Zhang P Qian T . Mir-129 controls axonal regeneration via regulating insulin-like growth factor-1 in peripheral nerve injury. Cell Death Dis. (2018) 9:720. doi: 10.1038/s41419-018-0760-1 29915198 PMC6006361

[115] Wang JL Huang QM Hu DX Zhang WJ . Therapeutic effect of exosomes derived from Schwann cells in the repair of peripheral nerve injury. Life Sci. (2024) 357:123086. doi: 10.1016/j.lfs.2024.123086 39357794

[116] Yang C Wang C Wang C Yang G Wu W . The effectiveness of platelet-rich plasma in the treatment of sciatic nerve injury: A single-blind randomized comparative trial. Neural Plast. (2025) 2025:7540054. doi: 10.1155/np/7540054 41306443 PMC12646722

[117] Khaled MM Ibrahium AM Abdelgalil AI El-Saied MA Yassin AM Abouquerin N . Efficacy of using adipose-derived stem cells and Prp on regeneration of 40 -mm long sciatic nerve defect bridged by polyglycolic-polypropylene mesh in canine model. Stem Cell Res Ther. (2024) 15:212. doi: 10.1186/s13287-024-03796-z 39020391 PMC11256418

[118] Mukai M Uchida K Miyagi M Inoue G Sekiguchi H Yokozeki Y . Fresh platelet-rich plasma gel reduces sciatic nerve-associated neuropathic pain in rats by suppressing tumor necrosis factor-A and interleukin-6 expression. Cureus. (2025) 17:e90019. doi: 10.7759/cureus.90019 40951078 PMC12431797

[119] Zhu Y Jin Z Wang J Chen S Hu Y Ren L . Ultrasound-guided platelet-rich plasma injection and multimodality ultrasound examination of peripheral nerve crush injury. NPJ Regener Med. (2020) 5:21. doi: 10.1038/s41536-020-00101-3 33298932 PMC7680141

[120] Pejkova S Stojkovski V Georgieva G Aleksovski B Tusheva S Srbov B . Platelet-rich plasma as a promising bioscaffold for enhancing peripheral nerve regeneration: An experimental study in a rat sciatic nerve model. J Biol Methods. (2025) 12:e99010051. doi: 10.14440/jbm.2025.0083 40200948 PMC11973046

[121] Esaki S Katsumi S Hamajima Y Nakamura Y Murakami S . Transplantation of olfactory stem cells with biodegradable hydrogel accelerates facial nerve regeneration after crush injury. Stem Cells Transl Med. (2019) 8:169–78. doi: 10.1002/sctm.15-0399 30417987 PMC6344901

[122] Júnior ED Valmaseda-Castellón E Gay-Escoda C . Facial nerve repair with epineural suture and anastomosis using fibrin adhesive: An experimental study in the rabbit. J Oral Maxillofac Surg. (2004) 62:1524–9. doi: 10.1016/j.joms.2004.05.216 15573353

[123] Cho HH Jang S Lee SC Jeong HS Park JS Han JY . Effect of neural-induced mesenchymal stem cells and platelet-rich plasma on facial nerve regeneration in an acute nerve injury model. Laryngoscope. (2010) 120:907–13. doi: 10.1002/lary.20860 20422684

[124] Sun Y Zhang R Mao X Zhang M . Research of acellular xenogeneic nerve combined with adipose-derived stem cells and platelet rich plasma in repair of rabbit facial nerve injury. Zhongguo Xiu Fu Chong Jian Wai Ke Za Zhi. (2018) 32:736–44. doi: 10.7507/1002-1892.201711079 29905054 PMC8414000

[125] Şahin MM Cayonu M Dinc SK Ozkocer E Ilhan M Uzunoğlu E . Effects of chitosan and platelet-rich plasma on facial nerve regeneration in an animal model. Eur Arch Otorhinolaryngol. (2022) 279:987–94. doi: 10.1007/s00405-021-06859-6 33956207

[126] Farrag TY Lehar M Verhaegen P Carson KA Byrne PJ . Effect of platelet rich plasma and fibrin sealant on facial nerve regeneration in a rat model. Laryngoscope. (2007) 117:157–65. doi: 10.1097/01.mlg.0000249726.98801.77 17202946

[127] Ismy J Khalilullah SA Maulana R Hidayatullah F . A potential treatment for erectile dysfunction: Effect of platelet-rich plasma administration on axon and collagen regeneration in cavernous nerve injury. Narra J. (2024) 4:e880. doi: 10.52225/narra.v4i2.880 39280316 PMC11391956

[128] Tai HC Tsai WK Chang ML Praveen Rajneesh C Tseng XW Hsu WC . Intracavernous injection of platelet-rich plasma reverses erectile dysfunction of chronic cavernous nerve degeneration through reduction of prostate hyperplasia evidence from an aging-induced erectile dysfunction rat model. FASEB J. (2023) 37:e22826. doi: 10.1096/fj.202201443R 36856608 PMC11977599

[129] Wu CC Wu YN Ho HO Chen KC Sheu MT Chiang HS . The neuroprotective effect of platelet-rich plasma on erectile function in bilateral cavernous nerve injury rat model. J Sex Med. (2012) 9:2838–48. doi: 10.1111/j.1743-6109.2012.02881.x 22906160

[130] Ding XG Li SW Zheng XM Hu LQ Hu WL Luo Y . Effect of platelet rich plasma on the regeneration of cavernous nerve: Experiment with rats. Zhonghua Yi Xue Za Zhi. (2008) 88:2578–80. 19080657

[131] Park GY Kwon DR . Platelet-rich plasma limits the nerve injury caused by 10% dextrose in the rabbit median nerve. Muscle Nerve. (2014) 49:56–60. doi: 10.1002/mus.23863 23558771

[132] Galán V Iñigo-Dendariarena I Galán I Prado R Padilla S Anitua E . The effectiveness of plasma rich in growth factors (Prgf) in the treatment of nerve compression syndromes of the upper extremity: A retrospective observational clinical study. J Clin Med. (2022) 11(16):4789. doi: 10.3390/jcm11164789 36013028 PMC9409748

[133] Malahias MA Johnson EO Babis GC Nikolaou VS . Single injection of platelet-rich plasma as a novel treatment of carpal tunnel syndrome. Neural Regener Res. (2015) 10:1856–9. doi: 10.4103/1673-5374.165322 26807124 PMC4705801

[134] Senna MK Shaat RM Ali AAA . Platelet-rich plasma in treatment of patients with idiopathic carpal tunnel syndrome. Clin Rheumatol. (2019) 38:3643–54. doi: 10.1007/s10067-019-04719-7 31420812

[135] Chen SR Shen YP Ho TY Li TY Su YC Chou YC . One-year efficacy of platelet-rich plasma for moderate-to-severe carpal tunnel syndrome: A prospective, randomized, double-blind, controlled trial. Arch Phys Med Rehabil. (2021) 102:951–8. doi: 10.1016/j.apmr.2020.12.025 33548206

[136] Shen YP Li TY Chou YC Chen LC Wu YT . Outcome predictors of platelet-rich plasma injection for moderate carpal tunnel syndrome. Int J Clin Pract. (2021) 75:e14482. doi: 10.1111/ijcp.14482 34107143

[137] van der Heijden RA Stewart Z Moskwa R Liu F Wilson J Hetzel SJ . Platelet-rich plasma for patellar tendinopathy: A randomized controlled trial correlating clinical outcomes and quantitative imaging. Radiol Adv. (2024) 1:umae017. doi: 10.1093/radadv/umae017 41059395 PMC12481695

[138] Estupiñan B Ly K Goldberg DJ . Adipose mesenchymal stem cell-derived exosomes versus platelet-rich plasma treatment for photoaged facial skin: An investigator-blinded, split-face, non-inferiority trial. J Cosmet Dermatol. (2025) 24:e70208. doi: 10.1111/jocd.70208 40414798 PMC12104007

[139] Holiuk Y Strafun S Lushchii O Ostapenko T Smorzhevskyi V . Development of classification and criteria of quality and safety of autologous platelet concentrates for regenerative injection therapy in traumatology and orthopedics. Cell Organ Transplantology. (2024) 12(2):76–83. doi: 10.22494/cot.v12i2.167

[140] Holiuk Y Strafun І . Development of a personalized approach concept for the application of regenerative technologies in patients with osteoarthritis and aseptic necrosis of the hip and knee joints. Cell Organ Transplantology. (2025) 13(1):4–12. doi: 10.22494/cot.v13i1.174

[141] Strafun S Holiuk Y Magomedov S Sokolov I Maslova T Ivanova O . Comparative analysis of clinical outcomes following regenerative injection therapy with autologous platelet concentrates in patients with partial rotator cuff tears. Cell Organ Transplantology. (2026) 14. doi: 10.22494/cot.v14-1.187

[142] Bansal H Leon J Pont JL Wilson DA Bansal A Agarwal D . Platelet-rich plasma (Prp) in osteoarthritis (Oa) knee: Correct dose critical for long term clinical efficacy. Sci Rep. (2021) 11:3971. doi: 10.1038/s41598-021-83025-2 33597586 PMC7889864

[143] Giusti I D'Ascenzo S Mancò A Di Stefano G Di Francesco M Rughetti A . Platelet concentration in platelet-rich plasma affects tenocyte behavior *in vitro*. BioMed Res Int. (2014) 2014:630870. doi: 10.1155/2014/630870 25147809 PMC4132404

[144] Jo CH Kim JE Yoon KS Shin S . Platelet-rich plasma stimulates cell proliferation and enhances matrix gene expression and synthesis in tenocytes from human rotator cuff tendons with degenerative tears. Am J Sports Med. (2012) 40:1035–45. doi: 10.1177/0363546512437525 22366517

[145] Han J Meng HX Tang JM Li SL Tang Y Chen ZB . The effect of different platelet-rich plasma concentrations on proliferation and differentiation of human periodontal ligament cells *in vitro*. Cell Prolif. (2007) 40:241–52. doi: 10.1111/j.1365-2184.2007.00430.x 17472730 PMC6496883

[146] Slapnicka J Fassmann A Strasak L Augustin P Vanek J . Effects of activated and nonactivated platelet-rich plasma on proliferation of human osteoblasts *in vitro*. J Oral Maxillofac Surg. (2008) 66:297–301. doi: 10.1016/j.joms.2007.05.022 18201612

[147] Mishra A Tummala P King A Lee B Kraus M Tse V . Buffered platelet-rich plasma enhances mesenchymal stem cell proliferation and chondrogenic differentiation. Tissue Eng Part C Methods. (2009) 15:431–5. doi: 10.1089/ten.tec.2008.0534 19216642 PMC2819709

[148] Berger DR Centeno CJ Steinmetz NJ . Platelet lysates from aged donors promote human tenocyte proliferation and migration in a concentration-dependent manner. Bone Joint Res. (2019) 8:32–40. doi: 10.1302/2046-3758.81.Bjr-2018-0164.R1 30800297 PMC6359887

